# Game-theoretic link relevance indexing on genome-wide expression dataset identifies putative salient genes with potential etiological and diapeutics role in colorectal cancer

**DOI:** 10.1038/s41598-022-17266-0

**Published:** 2022-08-04

**Authors:** Vishwa Jyoti Baruah, Papori Neog Bora, Bhaswati Sarmah, Priyakshi Mahanta, Ankumon Sarmah, Stefano Moretti, Rajnish Kumar, Surajit Borkotokey

**Affiliations:** 1 Centre for Biotechnology and Bioinformatics, Dibrugarh University, Dibrugarh, 786004 Assam India; 2 Department of Mathematics, Dibrugarh University, Dibrugarh, 786004 Assam India; 3 Department of Plant Breeding and Genetics, Assam Agricultural University, Jorhat, 785013 Assam India; 4 Centre for Computer Science and Applications, Dibrugarh University, Dibrugarh, 786004 Assam India; 5 CNRS, LAMSADE, Université Paris-Dauphine, PSL Research University, Paris, France; 6 Economics Group, Queen’s Management School, Queen’s University Belfast, Belfast, BT9 5EE UK

**Keywords:** Gene expression analysis, Cancer genomics, Gastrointestinal cancer, Tumour biomarkers, Biomarkers, Gastrointestinal diseases, Cancer genomics, Gastrointestinal cancer

## Abstract

Diapeutics gene markers in colorectal cancer (CRC) can help manage mortality caused by the disease. We applied a game-theoretic link relevance Index (LRI) scoring on the high-throughput whole-genome transcriptome dataset to identify salient genes in CRC and obtained 126 salient genes with LRI score greater than zero. The biomarkers database lacks preliminary information on the salient genes as biomarkers for all the available cancer cell types. The salient genes revealed eleven, one and six overrepresentations for major Biological Processes, Molecular Function, and Cellular components. However, no enrichment with respect to chromosome location was found for the salient genes. Significantly high enrichments were observed for several KEGG, Reactome and PPI terms. The survival analysis of top protein-coding salient genes exhibited superior prognostic characteristics for CRC. MIR143HG, AMOTL1, ACTG2 and other salient genes lack sufficient information regarding their etiological role in CRC. Further investigation in LRI methodology and salient genes to augment the existing knowledge base may create new milestones in CRC diapeutics.

## Introduction

Among the wide gamut of cancer types, colorectal cancer (CRC) marks its place as the third most recurrent type and seventh-most fatal disease/disorder globally^1,2^. Studies have shown that the prevalence of CRC increases with the person’s age^3^, though exceptional accounts of the disease occurrences in high proportion in the younger population have also been reported^3,4^. Global CRC occurrence can be majorly attributed (95% cases) to factors other than a person’s genetic predisposition and is a major contributor to cancer in developed and developing countries^1,3,5,6^.

Though the philosophy of studying diagnostics and therapeutics in an intertwined manner is not new, a common umbrella term ‘Diapeutics’ under the larger domain of cancer covering both aspects was long overdue until recently^7^. The tumors confined to the colon region metastasize to nearby lymph nodes in the absence of an early diagnosis. Treatment of CRC varies from medication, chemotherapy in early detection to surgery, excision and specific targeted therapeutics in severe cases of metastases to the liver or lungs^6,8–11^. Hence, early diagnosis and screening hold the key to reducing the incidence and mortality caused by the disease. The colonoscopy procedure is the most widely used diagnosis and screening strategy; however, it has several shortcomings, including being cost-ineffective, invasive, non-reliable and precarious^8–11^. On the other hand, biomarkers address these limitations by presenting a more cost-effective, reliable, and non-invasive early detection and screening technique of CRC^8,11^. Salient genes as biomarkers can provide with the ability of prediction (e.g. BRAF, ALK, ROS1, HER2, PI3K and miR-31-3p), prognosis (e.g. CIMP, CDX2 and MYO5B) and diagnosis (e.g. KRAS, p53, EGFR, erbB2)^12,13^.

Investigations on the tremendous amount of gene expression data generating specific patterns and gene co-expression networks and providing system-level functionality of genes have been effectively used to distinguish salient genes with the potential in diapeutics of a broad spectrum of complex human diseases^14^. The conventional data analysis methods on microarray take into account the down or upregulation of genes to find the salient genes. Only a few driver genes, when expressed aberrantly, are primarily responsible for the progression and advancement of the cancerous cell by bestowing the cell with a selective advantage in terms of either growth or delayed mortality^15,16^. However, a majority of differentially expressed passenger genes are not the causal factor and do not contribute to the overall initiation or progression of cancer, and their increase/decrease in gene expression is relatively co-incidental^15–18^. The conventional approach of designating deferentially expressed genes as the causative factor for complex human disease conditions raised several questions and doubts over the methodology^18^.

Microarray Network games have the potential to accurately depict the interactions among genes as their founding premise is to consider the interactions among players governed by a network structure^19–21^. In this study, the novel Game-Theoretic-Link Relevance Index (LRI) methodology^21^ is studied and applied to analyze the underlying salient genes and the associate functional annotations in CRC gene expression datasets. The resulting salient genes have an excellent potential in diapeutics, exhibiting characteristics essential for both diagnosis and therapeutics to mitigate this global complex human health condition. This work presents an opportunity to explore these salient genes further by experimental, preclinical, and clinical investigation to establish these as biomarkers.

## Materials and methods

### Colorectal cancer (CRC) patients dataset

For this study we used meta-dataset (E-MTAB-6698) from Arrayexpress database which comprises of 15 independent GEO datasets (GSE13067, GSE13294, GSE14333, GSE15960, GSE17536, GSE17537, GSE18088, GSE18105, GSE20916, GSE23878, GSE26682, GSE33113, GSE4107, GSE4183, GSE9348). All the GEO datasets of this meta-dataset were built using a common platform (GPL570; Affymetrix Human Genome U133 Plus 2.0 Array) to prevent deviations across different platforms. In brief, a total of 1566 underlying colorectal tissue samples microarray datasets, from tumor-free (control) and primary tumors (case), were preprocessed with RMA normalization, merged and ComBat-correction for batch effect correction. This large (meta-) dataset offers very high classifying accuracy (0.997) to test on TCGA (The Cancer Genome Atlas) dataset^22^ and serves as unparalleled cohort data for discovering salient genes crucial for disease phenotype development^23^.

### Estimation of differentially expressed genes (DEG) in the dataset

We assessed the microarray dataset using the conventional method of analysis. The ‘limma’ package^24^ from Bioconductor^25^ in R environment^26,27^ was utilized to identify DEGs as genes associated with CRC on a pre-normalized dataset (E-MTAB-6698). Linear models were applied and empirical Bayes statistics were calculated to assess DEGs between CRC samples and healthy control samples as defined by the designed experiments^28^. The genes with Benjamini–Hochberg False Discovery Rate (FDR) controlled adjusted p-value of ≤ 0.05 with Log2 Fold Change (LFC) ≥ 2 (two) were considered as DEG in CRC dataset^29^.

### Game-theoretic Link relevance Index (LRI) for co-expression network analysis

Herein, we utilized the CRC dataset and evaluated each gene using the LRI method^21^, as detailed in this section.

Let $(N,g^{E} )$ be a gene co-expression network where *N* represents a set of genes and $g^{E}$ be the set of links with respect to the Microarray Experiment Situation (MES) $E = < N;S_{D} ;S_{R} ;A^{{S_{D} }} ;A^{{S_{R} }} >$. Herein, $S_{D}$ and $S_{R}$ be the sets of samples from diseased and normal tissues, $A^{{S_{D} }}$ and $A^{{S_{R} }}$ be their expression matrices respectively. The link between *i* and *j* are in $g^{E}$ if two genes in *N* are co-expressed. The set of all links or edges, $g^{N} = \{ ij:i,j \in N,i \ne j\}$ is called the complete network. Let $G = \{ g:g \subseteq g^{N} \}$ denote the set of all possible networks on *N*. Let $N(g^{E} )$ be the set of players who have at least one link in $g^{E}$ i.e. $$N(g^{E} ) = \{ i:\begin{array}{*{20}c} {ij \in g^{E} for} & j \\ \end{array} \in N\}$$ and $n(g^{E} ) = |N(g^{E} )|$ denote the number of players involved in $g^{E}$. Given a $g^{E} \in G$, define the star of gene *i*, denoted by $g_{i}^{E}$, the set of links in $g^{E}$ that gene *i* is involved in i.e.$$g_{i}^{E} = \{ ij:ij \subseteq g^{E} \begin{array}{*{20}c} {for} & {j \in N(g^{E} )\} } \\ \end{array}$$. Degree of the node *i* is expressed as $|g_{i}^{E} | = d_{i} (g^{E} )$. Microarray network game $(g,v,g^{E} )$ was defined with the characteristic function $v:G \to R$ which assigns a worth to each set of link *g* representing the overall magnitude of the interaction between the genes. It follows that *v* determines the collective influence of a set of genes connected through a network based on their co-expression. It also follows that an equivalent form of the value function $v$ as a sum of unanimity games in a microarray network game $(g,v,g^{E} )$ is given by:

1 $$v(g) = \frac{1}{{n(g^{E} )}}\sum\limits_{{i \in N(g^{E} )}} {u_{{g_{i}^{E} }} } (g)$$

where the unanimity game $u_{{g_{i}^{E} }}$ is defined as:

2 $$u_{{g_{i}^{E} }} (g) = \left\{ {\begin{array}{*{20}c} 1 & {g_{i}^{E} \subseteq g} \\ 0 & {otherwise} \\ \end{array} } \right.$$

The value function $v$ specifies the total value that is generated by a given network structure. The class of microarray network games with player set *N* is denoted by $M^{N}$. The value function $v$ of the microarray network game $(g,v,g^{E} )$ picks up the information that can be used to define the role of each link in each co-expression of genes by applying suitable solution concepts of network games.

LRI allocates the total worth of the network among the genes. The allocation rule $F:G \times M^{N} \to R^{n}$ on the class of microarray network games $(g,v,g^{E} )$ is defined as:

$$F_{i} (g,v,g^{E} ) = \frac{1}{{n(g^{E} )}}\sum\limits_{\begin{subarray}{l} i \in N(g^{\prime}) \\ g^{\prime} \subseteq g \end{subarray} } {\frac{{|g_{i}^{\prime } |}}{{2|g^{\prime}|}}} \overline{\alpha }_{{g^{\prime}}} (v)$$

Here $\overline{\alpha }_{{g^{\prime}}} (v) = |\{ i \in N(g^{E} ):g_{i}^{E} = g\} |$. Thus if we take $g^{\prime} = g_{j}^{E}$ then $\overline{\alpha }_{{g^{\prime}}} (v) = 1$

3 $$F_{i} (g,v,g^{E} ) = \frac{1}{{n(g^{E} )}}\sum\limits_{{j \in N(g_{i}^{E} )}} {\frac{{|g_{i}^{\prime } |}}{{2|g_{j}^{E} |}}}$$

where $|g^{\prime}_{i} | = |\{ k \in N(g_{j}^{E} ):ik \in g_{j}^{E} \} |$, represents the number of genes associated with gene *i* i.e. the neighbourhood of gene i in $g_{j}^{E}$ and each gene *i* ∈ *N* receives half of the Shapley value of link $g_{i}^{\prime }$.

An equivalent form of the LRI is:

4 $$F_{i} (g^{E} ,v,g^{E} ) = \frac{1}{{2n(g^{E} )}}\left( {1 + \sum\limits_{{j \in N_{i} (g^{E} )}} {\frac{1}{{n_{j} (g^{E} )}}} } \right)$$

where $N_{i} (g^{E} ) = N(g_{i}^{E} )\backslash \{ i\}$ and $n_{j} (g^{E} ) = n(g_{j}^{E} ) - 1$. $N_{i} (g^{E} )$ denotes the set of neighbours of gene i in $g^{E}$ and $n_{j} (g^{E} )$ the numbers of neighbours of gene j.

LRI is a unique allocation rule which satisfies four desired properties, viz*.*,

- **anonymity** i.e. if $v(g_{1} ) = v(g_{2} )$ for all sub networks $g_{1} ,g_{2} \subseteq g$ such that they have same number of links i.e. $|g_{1} | = |g_{2} |$ then there exists $\alpha_{i} \in R$ for each *i* ∈ *N* such that $F_{i} (g,v,g^{E} ) = \alpha_{i} |g_{i} |$ for each link of microarray network game $(v,g^{E} ) \in M^{N}$,
- the **superfluous link property** i.e. $F(g,v,g^{E} ) = F(g\backslash ij,v,g^{E} )$ for all microarray network games $(v,g^{E} ) \in M^{N}$ and all links *ij* that are superfluous in $(v,g^{E} )$ i.e. those link which are not in $g^{E}$,
- **efficiency** which implies that $\sum\limits_{i \in N} {F_{i} (g,v,g^{E} } ) = v(g)$ for all network games (*N,v*) i.e. the sum of the relevance of all genes should be equal to the value of whole network, and
- **additivity** if $F(g,v_{1} + v_{2} ) = F(g,v_{1} ) + F(g,v_{2} )$, for each pair $(N,v_{1} ),(N,v_{2} )$ of network games with component additive value functions $v_{1}$ and $v_{2}$.

For example, consider the Microarray Experiment Situation, $E = < N;S_{D} ;S_{R} ;A^{{S_{D} }} ;A^{{S_{R} }} >$. $N = \{ 1,2,3,4\}$ be the set of genes and $g^{E} = \{ 12,13,14,23\}$ be the network on N. The value function *v* is such that

5 $$v(g) = \frac{1}{4}\{ u_{{\{ 12,13,14\} }} + u_{{\{ 12,23\} }} + u_{{\{ 13,23\} }} + u_{{\{ 14\} }} \} (g)$$

Which confers

6 $$v(g) = \left\{ {\begin{array}{*{20}l} 0 \hfill & {if\quad g = \{ 12\} ,\{ 13\} ,\{ 23\} ,\{ 12,13\} } \hfill \\ \frac{1}{4} \hfill & {if\quad g = \{ 14\} ,\{ 12,14\} ,\{ 12,23\} ,\{ 13,14\} ,\{ 13,23\} ,\{ 14,23\} } \hfill \\ \frac{1}{2} \hfill & {if\quad g = \{ 12,13,14\} ,\{ 12,13,23\} ,\{ 12,14,23\} ,\{ 13,14,23\} } \hfill \\ 1 \hfill & {if\quad g = g^{E} } \hfill \\ \end{array} } \right.$$

After calculation (using Eq. 1.3), the LRI of each gene for the microarray network game is $F(g,v,g^{E} ) = (\frac{18}{{48}},\frac{11}{{48}},\frac{11}{{48}},\frac{8}{48})$.

### Identification of the salient genes associated with CRC

We created an in-house script for calculating the LRI to the 4th decimal point. The genome-wide expression dataset with genes in rows and samples in columns was provided as the input matrix. With a large meta-dataset as input and the downstream analysis of the results, the script was executed on the AMD EPYC server with an AMD7301 processor and 256 GB memory. The resulting 126 salient genes with LRI values greater than zero were considered for downstream analysis. The salient genes were evaluated for distribution statistics and compared against all the known unique cancer biomarkers from the CellMarker database^30^ to ascertain the study’s uniqueness and novelty. The salient LRI genes were also compared against CRC DEG to ascertain the novelty in using LRI for salient gene discovery.

### Functional analysis of salient genes for CRC: biological network construction and enrichment analysis

To comprehend various biological roles that may be affected during the development of colon cancer, we tried to identify the ontologies from lists of 126 salient genes that were overrepresented. To avoid any changes in interpretations due to the evolution of the Gene Ontology and its annotations^31,32^, we updated the reference ontology library to the latest version. Overrepresentation of the Gene Ontology (GO) terms viz. biological process (BP), molecular function (MF), and cellular component were analyzed^33–35^. Right-sided Hypergeometric (enrichment) test with a cutoff p-value at 0.05, Benjamini–Hochberg p-value corrections, Kappa Score of 0.4 and a minimum of three (3) genes per cluster threshold was set to ascertain the enrichment.

We also checked the enrichment of 126 LRI genes in terms of location on the Chromosome to verify any biased expression of a particular locus/chromosome. The enrichment was performed for Chromosome location with 2025 terms/pathways with 61570 available unique genes (with the latest updated data of 17.02.2020) as reference data. Right-sided Hypergeometric (enrichment) test with a cutoff p-value at 0.05, Benjamini–Hochberg p-value corrections, Kappa Score of 0.4 and minimum of three (3) genes per cluster threshold was set to ascertain the enrichment.

To further evaluate the role of 126 salient genes in terms of the affected biochemical processes and identification of the critical reactions and pathways, enrichment analysis of Kyoto Encyclopedia of Genes and Genomes (KEGG) pathways, Reactome pathways and Reactome reactions (database updated latest on 08.05.2020) was performed with right-sided Hypergeometric (enrichment) test threshold p-value set at 0.05, Kappa Score of 0.4 and Benjamini–Hochberg p-value corrections. The obtained networks of the enriched biochemical pathways and reactions contain a variety of functional nodes and edges. All the functional enrichment analysis and visualization of the omics information was carried out as per the recommendation for standardization of the methodology^33–36^.

### Protein–protein interaction amongst the salient genes

To better visualize the role of 126 salient genes in providing functionality to a particular phenotype by interaction amongst them, we evaluate the extent of protein–protein interaction (PPI) among those genes. All the salient protein-coding genes were analyzed for PPI in the STRING database with high confidence (0.700) as threshold interaction score and all active interaction sources checked^37^. The isolated nodes were removed from the final result. The obtained networks of the enriched biochemical pathways and reactions contain a variety of functional nodes and edges. Various cluster terms were also evaluated, with Benjamini–Hochberg False Discovery Rate (FDR) less than 0.05 to identify the most significant PPI cluster.

Exploratory network analysis and statistics were evaluated using Cytoscape^33^ and R^27^.

### Survival analysis of the salient genes for CRC in TCGA data

Survival analysis of the protein-coding genes in CRC patients was evaluated using The Human Protein Atlas tool^38,39^. The CRC datasets available in the webserver contain mRNA expression levels of human genes from TCGA^40–42^of 597 cancer tissue samples from persons belonging to the alive/dead and male/female sub-group. The effect of the top 10 salient protein-coding genes on these samples was investigated for the overall survival endpoint. Herein, the expression values in FPKM of individual genes in different samples with their clinical outcomes are grouped into lower higher expressions based on median expression value. Log-rank test for Kaplan–Meier plot was utilized to assess these two groups for survival endpoints.

The effects of expression of the top 10 salient genes on patients’ survival across multiple CRC microarray datasets were retrieved from PrognoScan server^43^ to compare, assess and comprehend the novelty in the survival analysis of the genes in TCGA data^38,39^.

## Results and discussion

Biomarkers and targeted therapeutics were introduced for the early detection and clinical management of all cancer types, including CRC^7,44,45^. Therapeutics and cure of CRC include targeted medication in early diagnosis and chemotherapy and surgical resection in severe cases of metastases to other organs and tissues followed by medications^6,8–11^. Yet, recurrence of CRC in the presence of poor diagnostic measures was reportedly found to cause additional risk and reduce the life expectance of the people^2,7,9,10,44,46^ which can be attributed to widespread occurrences and recurrence of CRC, thus, making it one of the most dreaded diseases in the world^1–3,5,6,46^. Significant challenges were evident in successfully implementing specific biomarkers as a tool for cancer diapeutics^7,47,48^. Furthermore, despite several advances in cancer diapeutics, CRC continues to remain an unabated disease eventually leading to the death of the patient^1,2,5,6,49^. Therefore, a retrospection on our present knowledge on the factors with a prime etiological role in CRC is a must for mitigating the occurrence of CRC through targeted diapeutics.

Conventional algorithms for discovering genes of importance/biomarkers responsible for a physiological condition such as cancer rely on differentially expressed genes considered to be critical factors in the progression or manifestation of cancer condition. The conventional method identifies and prioritizes genes based on the degree of difference in expression values in cases (cancer) compared to control (normal healthy). These differentially expressed genes exhibit a high fold difference between gene expression values in cancer cases compared to normal conditions. In other words, the conventional methods convey that the genes with a greater degree of difference in expression level in disease samples than normal samples are more important than genes with a lesser degree of difference^18^. These methods possess many immediate challenges as the method dictates that the genes that exhibit greater fold difference in gene expression values are considered of greater importance, which may not be valid^18^.

Genes that initiate the cancer progression might have less fold difference in expression values than downstream effector genes, which often exhibit higher fold difference in expression^15–18^. Many passenger genes may exhibit greater fold difference though their contribution in the manifestation of the cancer is incidental^15–17^. On the other hand, driver genes, which are the causal factor, with comparatively lower fold difference in gene expression contribute more in the progression and advancement of the cancerous cell^15,16^. Also, these methods ignore the contribution of each gene in the overall gene network of cancer/case. Reassessment of diagnostic and prognostic markers for breast cancer and other cancer type were reported previously^18,50^. The investigators questioned the conventional approach and revealed that designating an etiological role in complex human disease conditions simply to the higher expressed genes may not be the correct methodology^18^.

Game theory (GT) has unlocked newer frontiers in solving various bioinformatics and computational biology challenges, from evolutionary genetics and virulence evolution modelling to high-throughput genomics data and biological networks^19,19–21,51–56^. Coalition GT on large-scale biological networks bestows estimation of the power of each gene governing biological pathways of interest and the associated etiological role in complex human health conditions^19,20^. GT application in quantitative evaluation of prominence of genes, by considering their relationships with others, in initiation and progression of disease condition contributed immensely in understanding the behaviors of salient genes in manifesting disease^54,55^. Cooperative Game theoretical approaches such as Shapley value and Banzhaf value provided valuable insight into gene expression data analysis by screening the dataset for the most relevant genes involved in the condition of interest^52,53^. Previously, the GT approach exhibited its ability at par with classical centrality indices in evaluating each gene by its relevance. It also emphasized the function of genes as nodes present in the periphery of a co-expression network in modulating the complex biological pathways^56^.

We adopted a Game Theoretic approach in this model, especially the approach of Network games. An improved GT method, LRI, was recently proposed to identify salient genes involved in cancer or other metabolic syndromes^21^. The LRI in this model is brought from the concept of Shapley value of cooperative game theory in networks which can be used as a relevant approach for the classification of genes^21^. A substantial attribute of LRI model of game theory is that it provides an innovative property-driven classification of the use of Shapley value as an index to validate and contextualize genes^57,58^. In microarray games, Shapley value was used to quantitatively evaluate the underlying genes involved in disease manifestation and characterise their role in gene-regulatory pathways^54,56,59^. LRI, on the other hand, utilizes a co-operative framework to analyze the microarray data of gene co-expression networks where genes and their connecting links play a significant role in determining the overall structure. It emphasizes that when we consider such a co-expression network, LRI can substitute Shapley value. This is because LRI focuses on the linking abilities of the genes as a suitable candidate to demonstrate the significance of the genes and is based on the position value (a link based allocation rule)^21^, while Shapley value is based on the Myerson value, which is a player based allocation rule^54,55^. LRI establishes that any network game can precisely describe the gene interactions as it considers the cooperation among genes and how the genes are connected to a network providing a comprehensive description of the genetic markers and their combined effects^21^.

E-MTAB-6698 is a large (meta-) dataset that comprises gene expression of colorectal tissue samples data with relevant clinical history and conditions of 1566 persons from both the biological gender. The whole-genome gene expression microarray data built on the GPL570 platform includes 121 colon samples from normal persons (as control), 1393 samples from the diseased part of the CRC patient, 37 samples from Adenoma patients, and 15 samples from patients suffering from Inflammatory bowel diseases. The overall dataset already proved its effectiveness by offering a very high degree of classification accuracy (0.997) to test the RNAseq dataset during training and modelling the disease condition. It functions as an unmatched cohort data for investigating and determining salient genes crucial for CRC development^22,23^.

Herein, we applied this game-theoretic LRI scoring^21^ on the high-throughput CRC transcriptomics dataset to identify salient genes in CRC. Contrary to the conventional approach, the Game-Theoretic Link relevance Index identifies a gene’s importance by considering genes’ contribution to overall disease manifestation.

We obtained 126 genes with a positive LRI score (LRI > 0) (refer to Supplementary File Table S1) which we referred as salient genes in the article. These 126 salient genes consist of 117 protein-coding genes, 8 non-coding RNA and 1 uncharacterized gene. Of these genes, four (4) were mapped on the X chromosome and the rest one hundred and twenty-two (122) on autosomal chromosomes. None of these 126 genes was mapped on the Y chromosome. Of these 126, the top 15 genes with the highest LRI score (Table 1) consist of one ncRNA and 14 protein-coding genes. *MIR143HG* and *AMOTL1* scored the highest LRI score (0.01604), followed by *ACTG2* (0.01587).

**Table 1:** LRI score of top 15 salient genes with their types and full name from nomenclature authority.

S. no	LRI score	Gene ID	Type of gene	Full name from nomenclature authority
1	0.016045	*MIR143HG*	ncRNA	Cardiac mesoderm enhancer—associated non—coding RNA
2	0.016045	*AMOTL1*	Protein-coding	Angiomotin like 1
3	0.015873	*ACTG2*	Protein-coding	Actin gamma 2, smooth muscle
4	0.011054	*FILIP1*	Protein-coding	Filamin A interacting protein 1
5	0.011054	*ARHGEF17*	Protein-coding	Rho guanine nucleotide exchange factor 17
6	0.011054	*FAM219B*	Protein-coding	Family with sequence similarity 219 member B
7	0.00959	*ITPKB*	Protein-coding	Inositol—trisphosphate 3-kinase B
8	0.00959	*TOP2A*	Protein-coding	DNA topoisomerase II alpha
9	0.00959	*HAND1*	Protein-coding	Heart and neural crest derivatives expressed 1
10	0.00959	*SERINC2*	Protein-coding	Serine incorporator 2
11	0.009081	*TRAP1*	Protein-coding	TNF receptor associated protein 1
12	0.009081	*CAMSAP1*	Protein-coding	Calmodulin regulated spectrin cassociated protein 1
13	0.009081	*APOBR*	Protein-coding	Apolipoprotein B receptor
14	0.009081	*PAG1*	Protein-coding	Phosphoprotein membrane anchor with glycosphingolipid microdomains 1
15	0.009081	*MRPS9*	Protein-coding	Mitochondrial ribosomal protein S9

**Figure 1:**
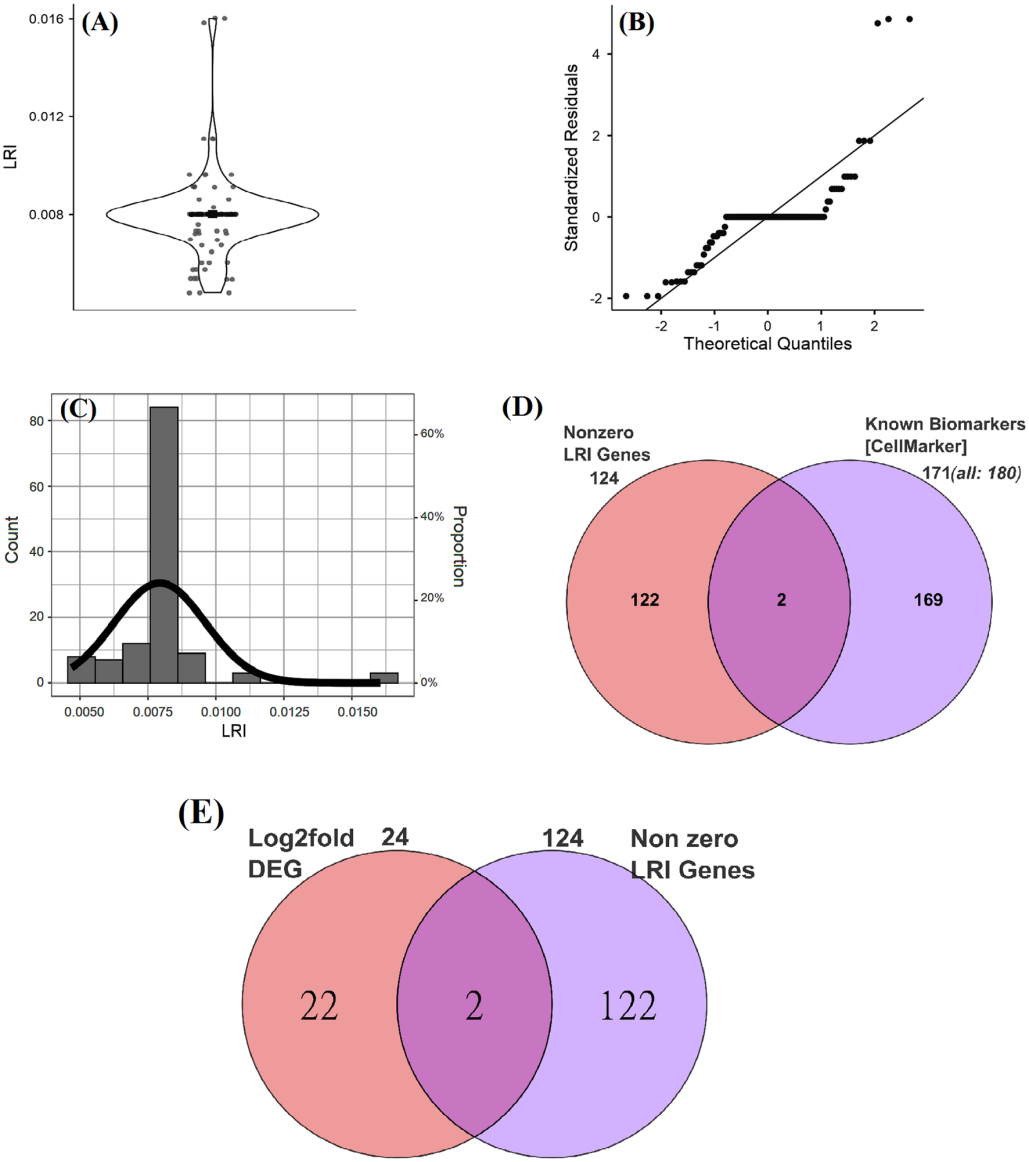
Distribution of LRI values of the salient genes. The figure exhibits the distribution of 126 salient genes. Violin plot (**A**) with jittered points data points and mean value, Q–Q plot (**B**) and histogram combined density plot (**C**) to show distribution LRI values of these 126 salient genes. (**D**) Comparison of the 124 salient genes (out of 126 Gene IDs, two Gene IDs did not map to Entrez ID) with 171 (after removing duplicates from the total 180 genes) unique cancer biomarkers from CellMarker database exhibits overlap of only two genes and 122 genes exhibits uniqueness. (**E**) Comparison of the 124 salient genes with 24 DEGs of CRC exhibits overlap of only two genes.

The distribution of the LRI value of 126 salient genes was analyzed to understand the nature of the data. Other genes with zero (0) LRI scores were not considered here for distribution study. A violin plot (Fig. 1A) depicts distributions of LRI values of 126 genes using density curves where the width of the curve specifies the approximate frequency of data points in that region. Quantile–quantile (Q–Q) plot (Fig. 1B) exhibits LRI data points falling in the middle of the plot and curve off in the extremities, indicating that the behaviors of the LRI data points are suggestive of high extreme values than would be expected if the data were normally distributed. The density-cum-histogram plot (Fig. 1C) describes the distribution of the LRI values of 126 genes against the count of genes (or proportion in secondary axis). All the exploratory data distribution analysis suggests that the distribution of LRI values is not normal and that disproportionate extremes of LRI values are assigned to those 126 genes.

CellMarker is an enormous curated database of biomarkers, especially at the single-cell level containing more than 22,000 cell markers of different cell types, including cancer cells^30^. To assess the uniqueness and novelty of the result in the present investigation, we extracted information of all the known biomarkers from the CellMarker database. Information of all the markers genes for cell types including Cancer cell, Cancer stem cell, Cancer stem-like cell, Tumor endothelial cell, and Tumor-propagating cell from the CellMarker database was retrieved. All the cell type individually provided information of 180 genes, which were further reduced to 171 after removing duplicates. We mapped 126 salient genes to Entrez ID for maintaining uniformity. One hundred twenty-four genes mapped to their corresponding Entrez ID except for two Genes IDs viz, *LOC100129461* and *LOC400965*. A comparative analysis (Fig. 1D) revealed that two genes, viz, *ITGA5*, and *MCAM* exhibited overlapped with the known biomarkers, suggesting that the information about these two genes is already present in the existing curated knowledge base cancer biomarkers. Except for these two, however, all the salient genes demonstrated no overlap with cancer biomarkers suggesting the resulting salient genes information exhibits novelty.

The conventional method of identifying genes associated with disease relies on the assumption that the greater the difference between the expression of the gene under the normal sample and the disease sample, the greater the chance that the gene is responsible for disease occurrence. The conventional method identifies the genes associated with CRC disease by isolating genes differentially expressed in the CRC sample compared to normal. The DEGs of CRC (Table 2) were compared to salient genes to ascertain the novelty in the LRI approach. The analysis (Fig. 1E) revealed that only two salient genes viz, *MT1M* (LRI value 0.007937), and *SI* (LRI value 0.007937) exhibited overlapped with the DEGs suggesting that the genes identified using the LRI approach is very much different from the conventional approach. These non-overlapping salient genes that do not overlap with the known biomarkers or with DEGs (Fig. 1D,E) present an opportunity to assess their role as diapeutics biomarkers further.

**Table 2:** DEGs (Differentially Expressed Genes) in the CRC dataset. The genes that exhibited adjusted p-value of ≤ 0.05 and Log2 Fold Change (LFC) ≥ 2 (two) were considered as DEG in the CRC dataset. DEGs that are also present in the list of salient genes are denoted by (*).

Genes	LFC	Average expression	t value	p-value	FDR adjusted p-value	B value
*GCG*	− 2.38358	4.969389	− 15.752	6.92E−52	1.29E−48	107.0804
*FOXQ1*	2.41793	9.809112	15.58703	6.45E−51	9.47E−48	104.8698
*CA1*	− 2.68165	7.905425	− 15.5739	7.71E−51	1.06E−47	104.6939
*CEMIP*	2.019388	8.943608	15.43317	5.10E−50	6.17E−47	102.8227
*CLDN8*	− 2.37615	4.975604	− 14.9159	4.78E−47	3.39E−44	96.04866
*AQP8*	− 2.20027	6.339491	− 14.6653	1.24E−45	6.68E−43	92.8299
*SLC4A4*	− 2.69512	6.884806	− 14.5002	1.03E−44	4.82E−42	90.73028
*PKIB*	− 2.09604	7.226978	− 13.9028	1.91E−41	5.58E−39	83.28646
MT1M*	− *2.12435*	*6.256335*	− *13.8111*	*5.93*E−*41*	*1.54*E−*38*	*82.16609*
*MS4A12*	− 2.71223	6.861364	− 13.7431	1.37E−40	3.35E−38	81.33784
*ZG16*	− 2.74676	8.079408	− 13.6444	4.59E−40	1.03E−37	80.14146
*CLCA4*	− 3.05432	6.704506	− 13.5764	1.05E−39	2.14E−37	79.32192
*CA2*	− 2.43465	8.684428	− 12.687	3.99E−35	4.53E−33	68.89684
*ADH1C*	− 2.11602	7.314942	− 11.9071	2.59E−31	1.71E−29	60.22568
SI*	− *2.37608*	*6.102779*	− *11.4662*	*3.02E−29*	*1.51E−27*	*55.52607*
*PCK1*	− 2.02539	8.368289	− 11.4555	3.38E−29	1.69E−27	55.41363
*MMP3*	2.000444	8.471801	11.2982	1.78E−28	8.17E−27	53.77398
*MMP1*	2.123265	8.813216	10.83644	2.09E−26	7.03E−25	49.07185
*KRT23*	2.006019	7.476861	10.55127	3.65E−25	1.08E−23	46.25175
*HEPACAM2*	− 2.06678	6.938537	− 10.292	4.65E−24	1.23E−22	43.74442
*CEACAM7*	− 2.10091	9.917928	− 10.0767	3.69E−23	8.83E−22	41.70331
*SLC26A3*	− 2.45108	9.32326	− 9.64708	2.06E−21	3.95E−20	37.74335
*ITLN1*	− 2.20724	7.95737	− 9.6134	2.80E−21	5.29E−20	37.43937
*CLCA1*	− 2.07261	8.930104	− 8.52471	3.65E−17	4.48E−16	28.12584

These non-overlapping genes present an opportunity to assess them further for their role as diapeutics biomarkers.

**Figure 2:**
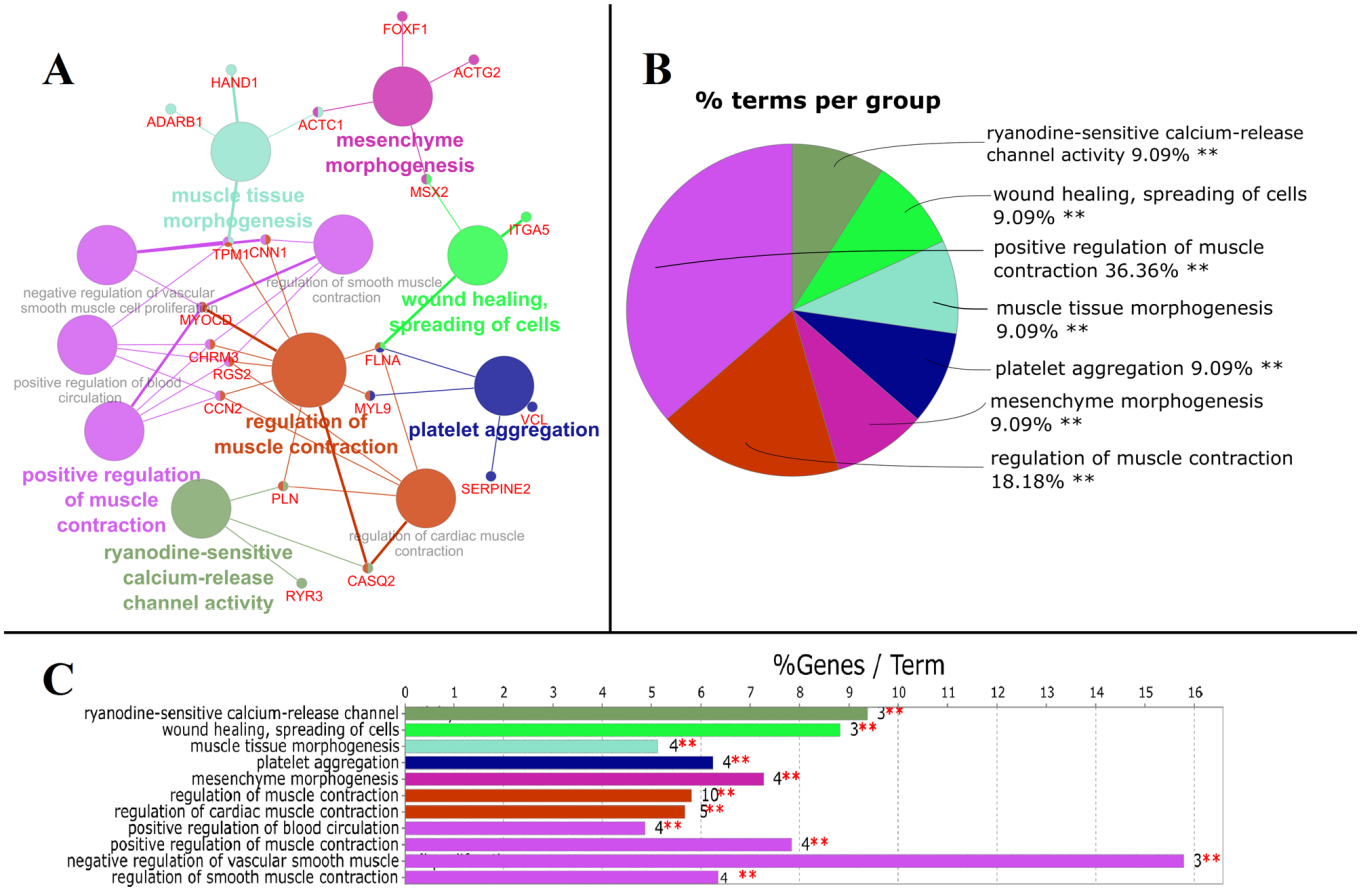
Enrichment of the major Biological Processes (BP) associated with the 126 salient genes. (**A**) Represents the network of various sub-ontologies, and associated genes, (**B**) describes percentage terms per group for various BP that are significantly enriched in pie chart and (**C**) number of genes in each term with significance sign. Node size is inversely proportioned to the *p*-value, i.e., the lower the value, the bigger the node size and color represent a different group of terms. *Significant at *p* ≤ 0.05, and **Significant at *p* ≤ 0.001.

The 126 salient genes were found to be associated with eleven Biological Process terms falling in seven GO groups (Fig. 2; Supplementary File Table S2), exhibiting overrepresentation. The enriched BP terms include ryanodine-sensitive calcium-release channel activity (GO:0005219), wound healing, spreading of cells (GO:0044319), muscle tissue morphogenesis (GO:0060415), platelet aggregation (GO:0070527), mesenchyme morphogenesis (GO:0072132), regulation of muscle contraction (GO:0006937), regulation of cardiac muscle contraction (GO:0055117), positive regulation of blood circulation (GO:1903524), positive regulation of muscle contraction (GO:0045933), negative regulation of vascular smooth muscle cell proliferation (GO:1904706) and regulation of smooth muscle contraction (GO:0006940). Ryanodine-sensitive calcium-release channel activity results in several skeletal myopathies due to dysregulation of intracellular Ca^2+^ and several muscle myopathies^60^. Wound healing and spreading of cells process is marked by collective migration of epithelial cells in the form of coherent sheets to heal wounds^61,62^. During cancer, one of the most prevalent phenomena is muscle dysfunction, where patients, irrespective of tumor stage and nutritional state, are subjected to compromised muscular function^63^. There have been several evidence that mitochondrial dysfunction can be induced by chemotherapy, which in turn contributes to muscle atrophy^64–68^. The biological process of tumor–induced platelet aggregation has several mechanisms involved which vary from tumor cell to the other and are generally activated by the generation of tumor cell-induced thrombin^69^.

**Figure 3:**
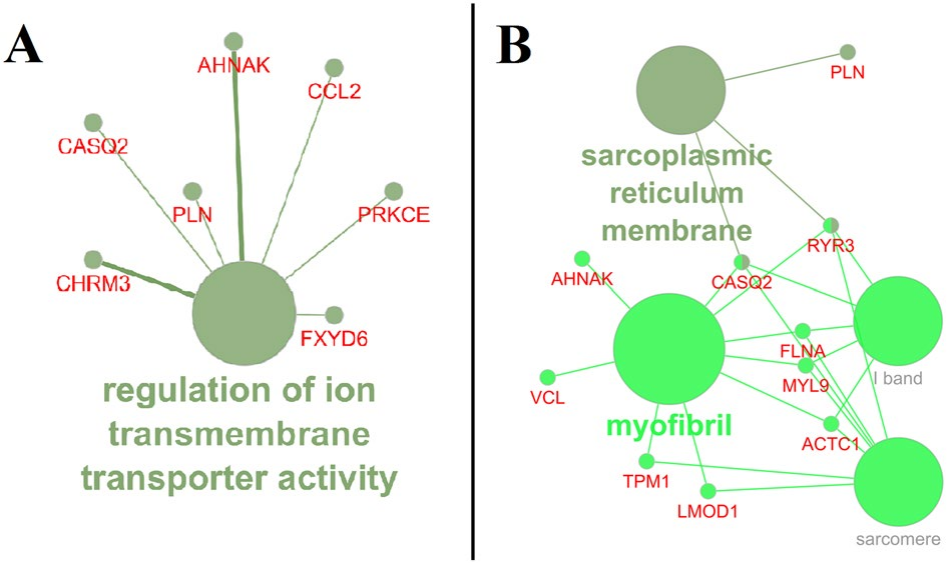
Enrichment of the major Molecular Function (MF) (**A**) and Cellular component (CC) associated with the 126 salient genes. (**A**) Describes percentage terms per group for various MF that are significantly enriched, and (**B**) shows various sub-ontologies of CC and associated genes. Node size is inversely proportional to the p-value, i.e., the smaller the value more considerable the node size and colour represents a different group of terms.

Furthermore, *AHNAK*, *CASQ2*, *CCL2*, *CHRM3*, *FXYD6*, *PLN*, and *PRKCE* genes are associated with the GO term for regulation of ion transmembrane transporter activity (GO:0032412) exhibited overrepresentation of the MF (Fig. 3A; Supplementary File Table S3). These genes may affect the progression of CRC as a consequence of perturbation of the critical process that modulates the activity of an ion transporter. This GO term demonstrated overrepresentation in a list of Cytosine—phosphate—Guanine (CpG) sites that exhibited a steady depolarization change^70^.

Overrepresented CC include Sarcoplasmic reticulum membrane (GO:0033017) with three associated genes (*CASQ2*, *PLN*, and *RYR3*), myofibril (GO:0030016) with nine (*ACTC1*, *AHNAK*, *CASQ2*, *FLNA*, *LMOD1*, *MYL9*, *RYR3*, *TPM1*, and *VCL*), sarcomere (GO:0030017) with seven (*ACTC1*, *CASQ2*, *FLNA*, *LMOD1*, *MYL9*, *RYR3*, *TPM1*), and I band (GO:0031674) with five associated genes (*ACTC1*, *CASQ2*, *FLNA*, *MYL9*, *RYR3*) (Fig. 3B; Supplementary File Table S4). *CASQ2* and *RYR3* are common genes between the Sarcoplasmic reticulum membrane (GO:0033017) and myofibril (GO:0030016) ontology terms. The Sarcoplasmic reticulum membrane has been associated with inherited dysfunctions and deficiencies like cardiac arrhythmias^71^. The enzymes involved in Sarco/endoplasmic reticulum calcium transport ATPases play a crucial role in loss or reduction of colon carcinomas and apoptosis^72^. Different myofibrils have been found to be associated with either oncogenic or tumor suppressor roles in different cancers like lung cancer, breast cancer, prostate and CRC^73^.

No enrichment with respect to chromosome location was observed for the 126 salient genes. These salient genes were observed to be distributed through the genome and not tandemly in a particular affected region. This suggests that the aberrant gene expression of the genes is not a consequence of the activation of a particular region in a chromosome.

**Figure 4:**
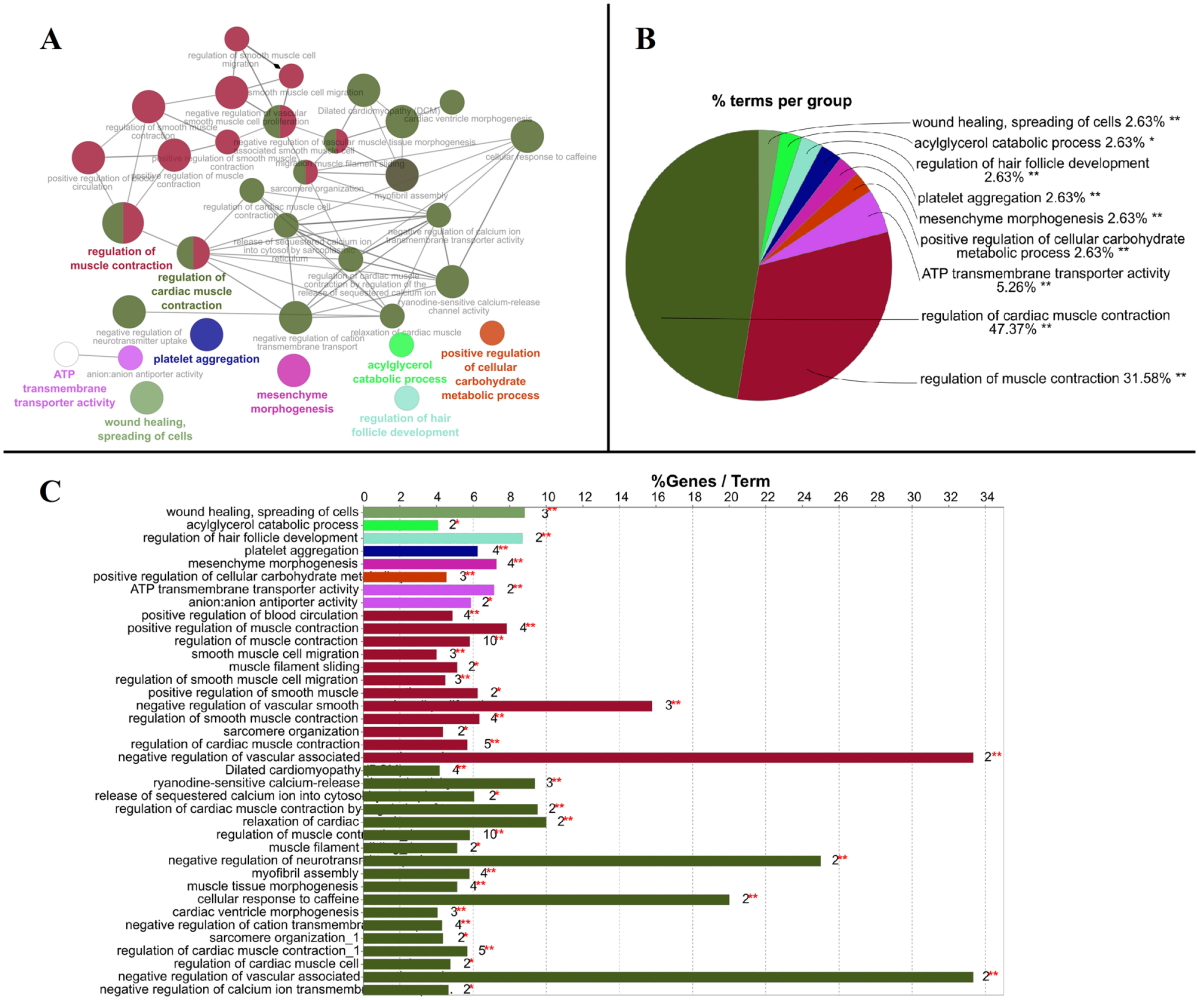
Enrichment of the major KEGG pathways associated with the 126 LRI genes. (**A**) Represents the network of various pathways and sub-pathways and associated genes where the size of the node is inversely proportional to the p-value, i.e., the lower the p-value, the bigger the node size and colour represents a different group of pathway terms. (**B**) Describes percentage terms per group for various parent pathways significantly enriched in pie chart, and (**C**) describes the number of genes in each pathway and sub-pathway term with significance sign. *Significant at *p* ≤ 0.05, and **Significant at *p* ≤ 0.001.

Thirty eight (38) KEGG pathway terms, viz., wound healing, spreading of cells (GO:0044319), acylglycerol catabolic process (GO:0046464), regulation of hair follicle development (GO:0051797), platelet aggregation (GO:0070527), mesenchyme morphogenesis (GO:0072132), positive regulation of cellular carbohydrate metabolic process (GO:0010676), ATP transmembrane transporter activity (GO:0005347), anion:anion antiporter activity (GO:0015301), positive regulation of blood circulation (GO:1903524), positive regulation of muscle contraction (GO:0045933), regulation of muscle contraction (GO:0006937), smooth muscle cell migration (GO:0014909), muscle filament sliding (GO:0030049), regulation of smooth muscle cell migration (GO:0014910), positive regulation of smooth muscle contraction (GO:0045987), negative regulation of vascular smooth muscle cell proliferation (GO:1904706), regulation of smooth muscle contraction (GO:0006940), sarcomere organization (GO:0045214), regulation of cardiac muscle contraction (GO:0055117), negative regulation of vascular associated smooth muscle cell migration (GO:1904753), Dilated cardiomyopathy (DCM) (KEGG:05414), ryanodine-sensitive calcium-release channel activity (GO:0005219), release of sequestered calcium ion into cytosol by sarcoplasmic reticulum (GO:0014808), regulation of cardiac muscle contraction by regulation of the release of sequestered calcium ion (GO:0010881), relaxation of cardiac muscle (GO:0055119), regulation of muscle contraction (GO:0006937), muscle filament sliding (GO:0030049), negative regulation of neurotransmitter uptake (GO:0051581), myofibril assembly (GO:0030239), muscle tissue morphogenesis (GO:0060415), cellular response to caffeine (GO:0071313), cardiac ventricle morphogenesis (GO:0003208), negative regulation of cation transmembrane transport (GO:1904063), sarcomere organization (GO:0045214), regulation of cardiac muscle contraction (GO:0055117), regulation of cardiac muscle cell contraction (GO:0086004), negative regulation of vascular associated smooth muscle cell migration (GO:1904753), and negative regulation of calcium ion transmembrane transporter activity (GO:1901020) exhibited significantly high enrichment (Fig. 4; Supplementary File Table S5). Acylglycerol catabolic process has been used as a biomarker for the diagnosis and/or prognosis of CRC, and the enzymes of acylglycerols are involved in CRC tumor growth survival and metastasis^74^. Changes in the cellular carbohydrate metabolic process may precede the acquisition of driver mutations, ultimately leading to colonocyte transformation. These changes may not be uniform but rely on different pathways to adapt to nutrient availability^75^. Muscular contraction was found to be enriched in the signal pathway of the differentially expressed genes associated with the early onset of CRC^29^.

**Figure 5:**
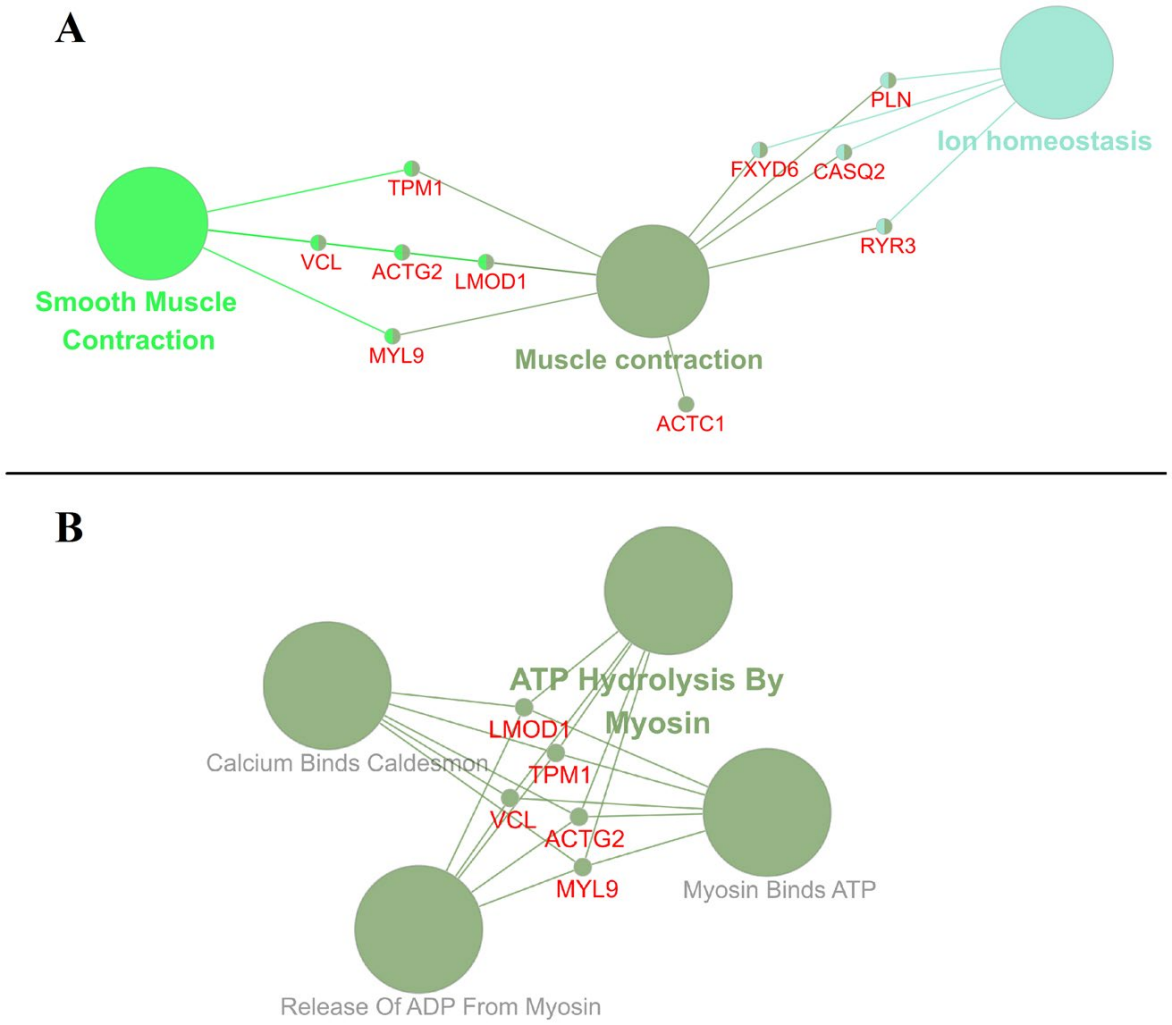
Enrichment of the major Reactome pathways and reactions. The figure represents the Enrichment of the Reactome pathways (**A**) and reactions (**B**) associated with the 126 salient genes (Labeled in red colour). Node size is inversely proportional to the p-value, i.e., the lower the p-value, the bigger the node size and colour represents a different group of terms.

We searched for enriched Reactome pathways using all available genes as a reference from the database. Three terms viz. Muscle contraction, Smooth Muscle Contraction, Ion homeostasis exhibited high significant enrichment (Fig. 5A; Supplementary File Table S6). All the three Reactome pathway terms revealed equal enrichment with 33.33% of the total terms distributed per group. Five (5) genes, viz. *ACTG2*, *LMOD1*, *MYL9*, *TPM1*, *VCL* are associated with Smooth Muscle Contraction (R-HSA:445355), four (4) genes viz. *CASQ2*, *FXYD6*, *PLN*, *RYR3* are associated with Ion homeostasis (R-HSA:5578775) and ten (10) genes, viz, *ACTC1*, *ACTG2*, *CASQ2*, *FXYD6*, *LMOD1*, *MYL9*, *PLN*, *RYR3*, *TPM1*, *VCL*, are associated with Muscle contraction (R-HSA:397014). Of the 126 salient genes of CRC, these genes are primarily associated with muscle contraction. Their aberrant expression must affect the associated processes and functional proteins in the progression of CRC. Muscle contraction and dysfunction have been found to be intensely associated with CRC, and their respective molecular functions indicate that they could possibly be the therapeutic targets of CRC^29,76^. Their aberrant expression must affect the associated processes and functional proteins in the progression of CRC. Ion homeostasis plays an indispensable role in the physiology of the gastrointestinal tract, and any dysregulation is an indication of gastrointestinal cancer. They can, therefore, be used as a useful prognostic biomarker for gastrointestinal cancer^77^. Cancer metastasis has often been found to be accompanied by skewing in ion homeostasis^78^.

We also searched for enriched Reactome reactions using all available genes as a reference from the database. Five (5) genes viz. *LMOD1*, *TPM1*, *VCL*, *ACTG2*, and *MYL9* contributed towards very high significant enrichment (100% of the terms per group) exhibited by the term ‘ATP Hydrolysis By Myosin (R-HSA:445699)’ (Fig. 5B; Supplementary File Table S7). With Kappa Score of 0.4, which is used to define term-term interrelations (edges) and functional groups based on shared genes between the terms, Reactome reactions viz. ATP Hydrolysis by Myosin (R-HSA:445699), Myosin Binds ATP (R-HSA:445700), Calcium Binds Caldesmon (R-HSA:445704), Release of ADP From Myosin (R-HSA:445705) exhibited high enrichment suggesting that the salient genes majorly associated with smooth muscle contractions activity. Results obtained recently also suggest that the Muscle contraction and vascular smooth muscle contraction pathway as major affected molecular mechanisms in CRC^29^, which corroborate the inference of our present work.

**Figure 6:**
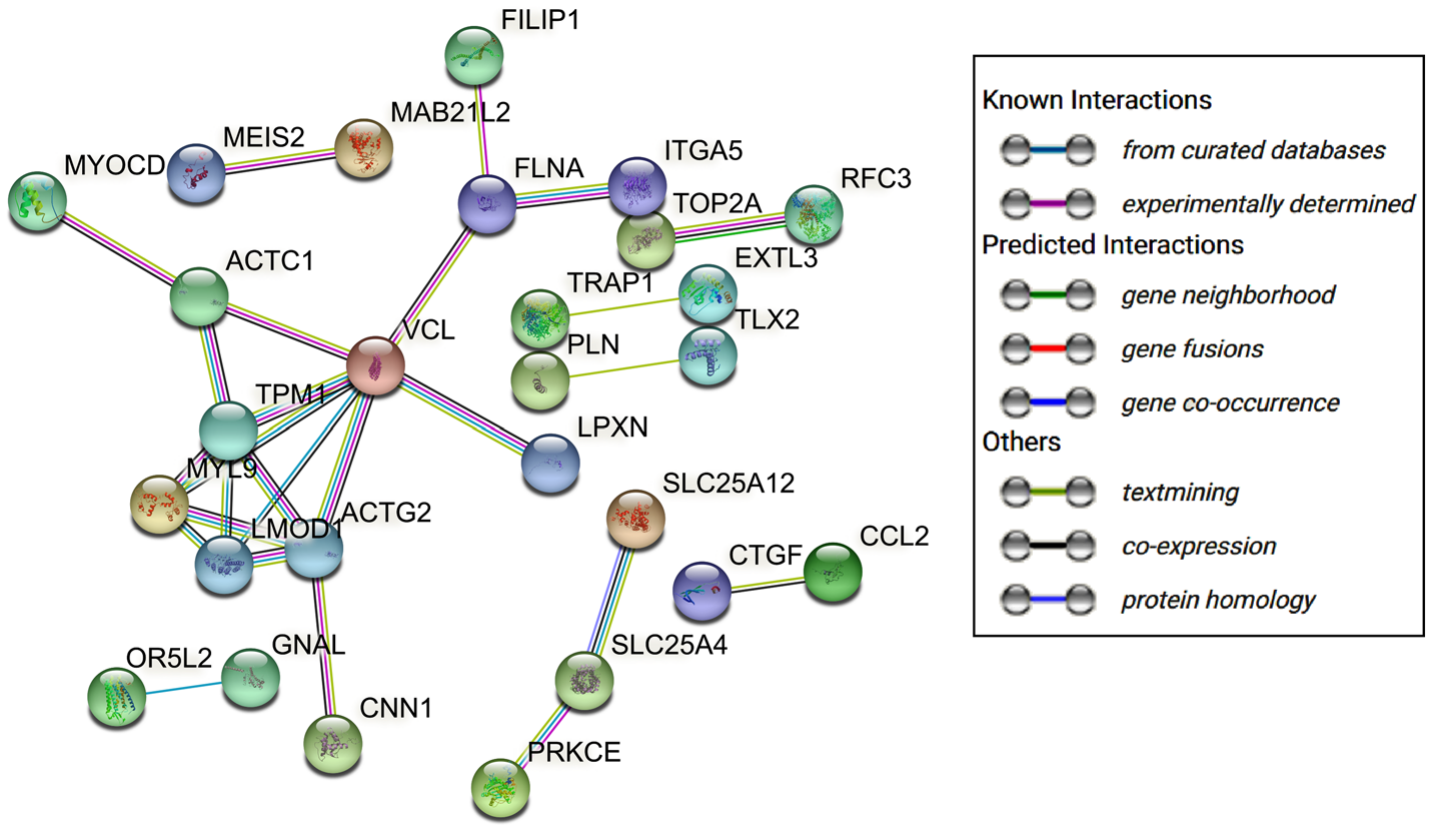
Protein–Protein interaction (PPI) network among the 126 salient genes. At a confidence score of 0.700, the figure exhibits major interactions among the protein-coding genes as node and various interactions as edges. The colored nodes are query proteins with 3D structures (if any) inside the node and edge color represents evidence as an indicator of interactions among the proteins. The isolated nodes were removed from the network.

We investigated protein–protein interaction (PPI) among the salient genes (Fig. 6). STRING database^37^ was able to identify 116 protein-coding genes as nodes and presented the PPI network. At a threshold confidence score of 0.700 (high confidence score), a total of 26 edges were formed with an average node degree equal to 0.448, the average local clustering coefficient of 0.198. With expected number of edges equals 9, the network exhibited significant enrichment (p-value = 0.00000141 < 0.05). Total ten clusters were found with FDR p-value less than 0.05 suggesting these clusters are significantly enriched (Supplementary File Table S8 The most prominent clusters, CL:1326 and CL: 1328, consist of 10 and 8 protein-coding genes, respectively, and are associated with ‘Muscle protein and Myofbril assembly’. The smallest clusters, CL:25786, CL:1449, CL:1577, CL:6451, consist of only two protein-coding genes each. All the 10 clusters exhibited cluster value greater than 1 for Strength which is a measure of enrichment effect and is expressed as Log10 value of the ratio between observed gene count in the network and expected gene count suggesting and value lesser than 0.05 for FDR, suggesting the results of high enrichments are statistically significant (Supplementary File Table S8).

It is also pertinent to note that some in vitro and in vivo reports suggest the top three genes viz. *MIR143HG*, *AMOTL1*, and *ACTG2* may be associated with other cancer subtypes. However, after a thorough literature survey, we were not able to mine any data suggesting their etiological role in CRC development.

MIR143HG (LRI = 0.016045) is a highly conserved long non-coding RNA that hosts miR-143/145 cluster that modulates smooth muscle cell differentiation and remodelling^79^. The association of the MiR143HG with bladder cancer and hepatocellular carcinoma is well established^80,81^. MiR143HG/MIR-1275/AXIN2 tri-axis is directly responsible for the onset of bladder cancer its progression by tempering the Wnt/β-catenin pathway. Its downregulation is directly associated with the development and progression of bladder cancer^80^ while an upregulation is associated with hepatocellular carcinoma^81^. It also has an important functional role(s) in cardiovascular system development^79^.

AMOTL1 (LRI = 0.016045) is a motin family member or Angiomotins (AMOTs) that dictates the functioning of several bioprocesses, including tight junction formation, angiogenesis, cell polarity, and migration^82,83^. Previous reports suggest *AMOTL1* is an oncogene and their dysregulated expression affects promotion, proliferation, migration and relapse of cancer cells, including prostate cancer, renal cell cancer, cervical cancer, liver cancer, head and neck squamous cell carcinoma, bladder cancer, and osteosarcoma^82–85^. Contrary to this, it also exhibits tumor suppression function inhibiting cancer cells’ growth in glioblastoma, ovarian cancer, and lung cancer^82^.

The actin gamma smooth muscle 2 (*ACTG2*) gene ((LRI = 0.015873), belonging to the actin protein family, is imperative for maintaining the cytoskeleton through the regulation of cell movement and muscle contraction^86^. Genome sequencing studies have revealed that a homozygous and a heterozygous variant of *ACTG2* is associated with gastrointestinal dysfunction^87^. Studies have demonstrated that the over-expression of *ACTG2* has been found to play a critical role in the progression of hepatocellular carcinoma^88^ and bladder cancer^89^. Conversely, another finding has described a concomitant improved survival and more aggressive phenotype with a higher expression of *ACTG2*^90,91^. However, a lower expression of the gene was associated with normal colon tissue in contrast to colon carcinoma^92^, while imperceptible expression levels of *ACTG2* have been associated with the metastasis of lymph nodes^93^. A recent bioinformatics work comparing samples of 12 CRC patients and 10 healthy control group also hinted at the possible role of *ACTG2* in manifesting CRC^29^, which is consistent with our findings.

Filamin A interacting protein 1 (*FILIP1*), a potent antivascular cancer therapeutic, has been demonstrated previously to be a key modulator of angiogenesis’s inhibitory effects^94^. Moreover, the *FILIP1* is also found to inhibit cell invasion and metastasis in ovarian cancer by downregulating the Wnt pathway^95^. On the other hand, the Rho guanine nucleotide exchange factor 1 protein (*ARHGEF17* gene), formerly known as a guanine nucleotide exchange factor (*GEF*), is a vital mitotic gene. ARHGEF17 is indispensable for the spindle assembly checkpoint and targets mitotic kinase Mps1 to mitotic kinetochores^96^. It is also presumed to be responsible for lung carcinoma cell migration stimulated with lysophosphatidic acid^97^. The Family with Sequence Similarity 219 Member B (*FAM219B*), a paralog of *FAM219A* gene, is a protein-coding gene. The diseases associated with it include Metachondromatosis and Leopard Syndrome 1^98,99^.

The information on the role of the top 10 genes according to LRI in CRC development is either not present or is negligible. Our data mining suggests the unavailability of any previous information indicating the role of *FAM219B* in any cancer type. Notwithstanding the potential role of salient genes in other cancer types, the central role of *MiR143HG*, *AMOTL1*, *ACTG2*, *FILIP1*, *ARHGEF17*, *FAM219B* in the progression of CRC is inadequate and lacking in previous reports. Previous reports on *TOP2A*^100,101^, *ITPKB*^101^, *HAND1*^102^, *SERINC2*^103–105^ present a conjectural view of the importance of the gene in the progression of the CRC.

**Figure 7:**
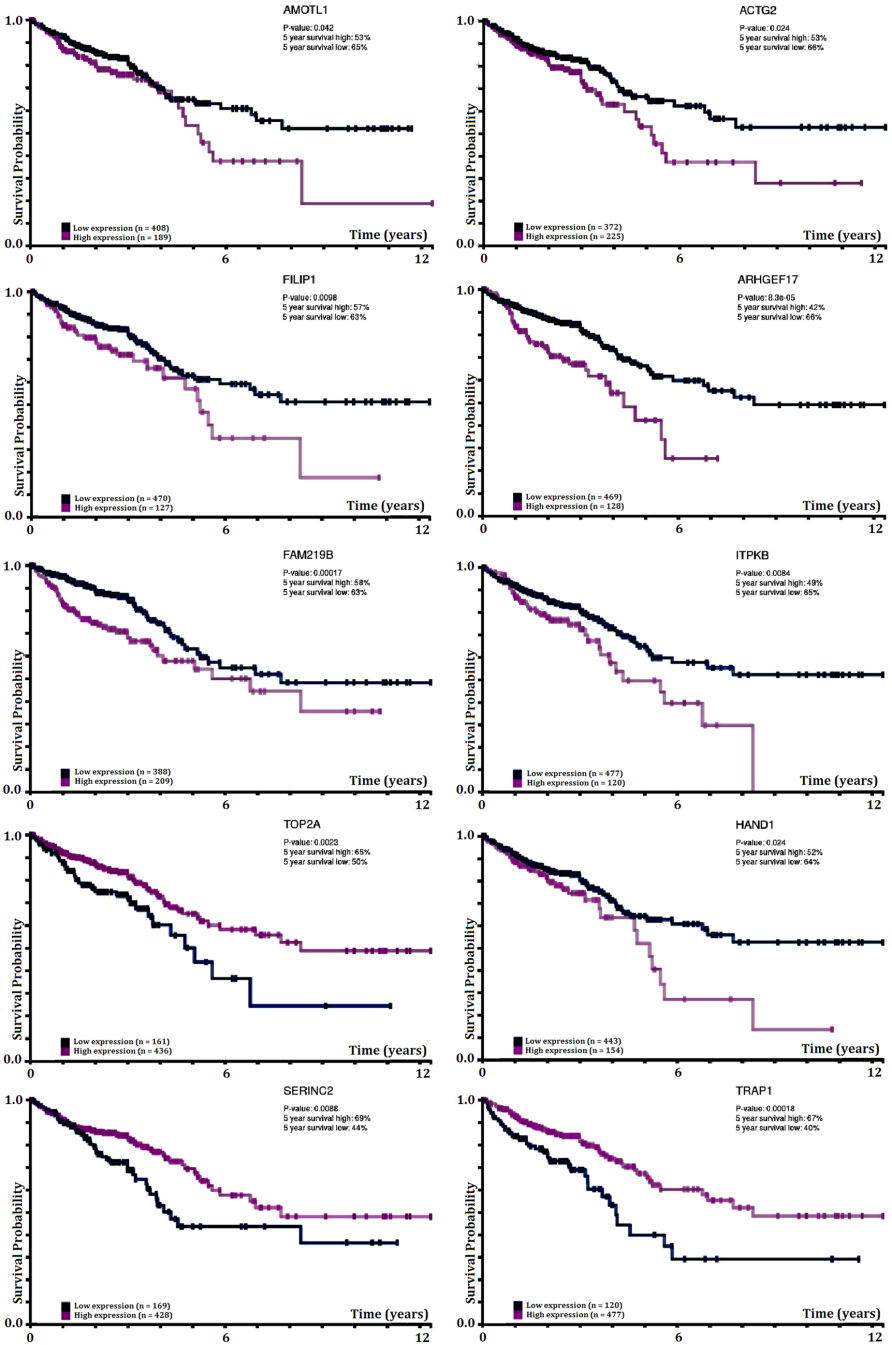
Diapeutics implication of top 10 protein-coding salient genes in CRC. Kaplan–Meier (KM) survival analysis of overall survival with respect to expression of top 10 protein-coding salient genes in CRC samples. In each plot, the abscissa represents ‘Time in Years’ and the ordinate represent ‘Survival Probability’. Log-rank p-value for KM plot represents a significant correlation between mRNA expression level and patient survival by exhibiting significant differences in survival between genes’ high and low expression. Protein-coding salient genes exhibited statistically significant (log-rank p-value ≤ 0.05) in the overall survival endpoint (refer to Supplementary File Figure S1–S10 for enlarged view).

Identifying the top salient genes as diapeutics biomarkers for CRC will be critical to diagnostics, predicting the disease’s occurrence/recurrence, and improvising the therapeutics. Major statistical difference in weak expression and high expression of genes in Kaplan–Meier (KM) survival analysis highlights the importance of genes with respect to their significant contribution to cancer progression and development^50^. The top 10 protein-coding genes were investigated for patients’ survivability and their expression (Fig. 7). Log-rank p-value for KM plot indicates a correlation between patient’s survival and gene expression level. Statistically significant results (log-rank p-value ≤ 0.05) in the overall survival endpoint suggest that the change in expression of genes compared to the cutoff threshold at the molecular level results in a notable difference in the overall patient’s survival probability. The top 10 salient genes expression exhibited statistically significant results (log-rank p-value ≤ 0.05), connoting the high significance of these genes in causing CRC progression and development during perturbed expression. There exists a discernible difference in 5-year survival for patients with lower expression compared to higher expression than their respective expression cutoff for a 5-year duration. The median survival time between lower and higher expression of the genes are significantly different. Among the top 10 salient protein-coding genes, *AMOTL1*, *ACTG2*, *FILIP1*, *ARHGEF17*, *FAM219B*, *ITPKB*, *HAND1* exhibited reduced survival probability with higher expression, contrary to the rest of the other genes, viz. *TOP2A*, *TRAP1*, *SERINC2*, which exhibited reduced survival probability with lower gene expression (Fig. 7, also refer Supplementary File Figure S1–S10 for enlarged view).

All the genes exhibit a distinct difference in the survival endpoints between the two expressions. The KM plot of the top 10 protein-coding salient genes (highlighted by the LRI score) revealed a significant contribution to the CRC patients' overall outcome. A survey of these top 10 salient genes in PrognoScan, a server to search relationship between expression of genes and patients’ overall and disease-free survival across an ensemble of microarray datasets^43^, revealed variation in the results between the microarray datasets (Supplementary spreadsheet). This variation in the results can be attributed (to some extent) to sampling error owing to the small-scale nature of the microarray dataset. Moreover, as most of these genes do not qualify characteristics of a DEG (t-test adjusted p-value of  ≤ 0.5 and Log Fold Change more than 2), they are not considered to be associated with the manifestation of disease outcome when applying conventional microarray analysis technique.

MalaCards^106^ is a comprehensive human disease/maladies database integrating data from more than seventy sources, including GeneCards^107^ and GeneAnalytics^108^. It provides ‘MalaCards InFormaTion (MIFT) Score’, which signifies the richness of the gene’s information against each disease associated with it; the higher the MIFT score, the more significant the annotation results of the gene is to the disease based on previously published literature^106^. The top 15 genes with the highest LRI score were further evaluated in the MalaCards database^106^ to assess their annotation of the disease to the Gene to verify the significance of the results (Supplementary File Table S9).

Though varying MIFT score against major cancer is evident from multiple reports on the involvement in major cancer type, seven (7) genes viz*.* *MIR143HG*, *AMOTL1*, *FILIP1*, *FAM219B*, *SERINC2*, *APOBR*, *MRPS9* exhibited no MIFT score and no results against CRC (aqua blue rows in Table S9 of Supplementary File). Another seven (7) genes viz. *TRAP1*, *ACTG2*, *HAND1*, *ITPKB*, *PAG1*, *CAMSAP1*, *ARHGEF17* are known to be associated with other cancer types as evident by the existence of prior reports on these genes; yet they exhibited low MIFT score against CRC, suggesting these genes lacks sufficient annotated information on the genes’ involvement in CRC owing to the scanty number of the report as strong conclusive evidence to corroborate (olive green rows in Table S9 of Supplementary File). The result implies the novelty in the present work as none-to-scanty reports exist for most top genes in terms of association with CRC. Only *TOP2A* exhibited a high MIFT score against CRC and other cancer types, suggesting strong evidence of prior report on the association with CRC and other cancer types (purple rows in Table S9 of Supplementary File). The *TOP2A* being in the top LRI scoring gene and exhibiting a high MIFT score also suggest that the present work is endorsed by the corroborating work published previously (Supplementary File Table S9). All the genes exhibited a varying degree of results in terms of association with other cancer types.

Previously, graph theory-based work demonstrated using the Human Disease Network and Disease Gene Network that majority of the molecular machinery underlying diseases are highly interconnected– sharing functionally^109,110^ as well as their genomic changes^111^. The nature of upstream perturbation in the activity of genes interconnected in a network of complex metabolic pathways can relay diverse perturbation effects in the dynamics of downstream functionality, which may lead to any of the diverse range of (patho) phenotypes associated with downstream pathway’s activities^110^. A glance over the MalaCards table reveals that most of these top genes are involved in other cancer subtypes, including breast cancer. A high degree of interconnectedness in genes is observed in CRC with breast cancer and lymphoma. Moreover, both CRC with breast cancer shares many genes with the etiological role^109^. Thus, it is apparent that genes with no or low MIFT score for CRC also exhibited no or low scores for Breast cancer and genes that exhibited high MIFT scores for CRC also demonstrated high scores for Breast Cancer (Table S9 of Supplementary File). Prior reports exhibited that many of these genes are differentially expressed and have a possible etiological role in the manifestation of CRC and Breast Cancer (compared to normal conditions). However, the regulation patterns of these genes in combination (upregulation or downregulation) with respect to CRC and Breast Cancer are not synchronized and lacks coherence^112^. Moreover, the cancer genome ‘landscape’ suggest varying degree of acquisition of mutation that drives the cell towards CRC or Breast cancer^113^.

The LRI method ranks the genes relevance to the condition in an asymmetrical way, with 126 salient genes exhibiting the positive LRI score. The top 10 genes exhibited LRI scores in quartile above the median rank score for these 126 genes (Fig. 1). The extreme values assigned to these genes suggest that their relevance compared to other lower-ranked genes is relatively more, and the relevance of these lower-ranked genes is more than other genes not included in the (Supplementary File Table S1) list. It is well-established that the genes clustering together in expression analysis exhibit common biological ontologies^18,50,114,115^. Hence, predominant functions associated with all the salient genes in a specific cellular context can provide a clear view of affected functional terms in CRC progression. Various GO (Figs. 2 and 3), KEGG (Fig. 4), Reactome (Fig. 5) and PPI (Fig. 6) terms exhibited over-representation than normal in CRC samples characterized by statistically significant low p-value. These salient genes’ similar scores can be attributed to the various networks they represent. The significance of the methodology is evident from the observation that relevance of the top 10 protein coding genes as major player with probable etiological role in CRC was also clearly established by the using Kaplan and Meier method of survival analysis (Fig. 7) with significance assessment using Log-Rank tests^50,116^. The novelty of the method can be assessed from the fact that majority (except two) of the salient genes are absent in the existing knowledge database of cancer biomarkers (Fig. 1D). The resulting salient genes showed a dearth of the previous reports in a highly cited and manually curated biomarkers database of repute^30^. The report opens up new dimensions in investigating these salient genes by in vitro and in vivo experiments and ushers new hope in diapeutics by providing novel gene targets for mitigating the development and metastasis of CRC. The report also stresses the algorithm’s effectiveness in assessing the importance of individual genes in cancer etiology, utilizing only expression patterns at the molecular scale. This report also presents an opportunity to pounder over the use of non-conventional GT approaches in assessing genes’ relevance using genome-wide expression dataset for application in diapeutics to conquer this and other dreaded diseases.

Finally, we can conclude that the mortality caused by cancer can be checked by early diagnostic screening with the help of biomarkers and effective targeted therapeutics. The knowledge discovery of salient genes associated with CRC can fill many voids related to biomarkers, perturbed biochemical pathways, and genes’ action during and prior to cancer development. We employed a game-theoretic link relevance Index (LRI) scoring approach on the high-throughput transcriptomics dataset to identify salient genes in CRC. One hundred and twenty-six (126) salient genes demonstrated a positive LRI score (LRI > 0), indicating the significance of these genes in network games of genes. Investigation of the diverse gene ontology revealed eleven overrepresentations for major Biological processes. GO term for regulation of ion transmembrane transporter activity (GO:0032412) exhibited overrepresentation of the Molecular Function while six overrepresentations were obtained for major Cellular Component. Although considerable enrichment was observed for thirty-eight KEGG pathways and three Reactome pathways, no enrichment was observed for the salient genes concerning chromosome location. The investigation reports the centrality nature of *MiR143HG*, *AMOTL1*, *ACTG2*, *FILIP1*, *ARHGEF17*, *FAM219B*, and other genes in CRC progression, which is lacking in previous studies and public repositories. Furthermore, the resulting information will enhance and supplement the existing knowledge base on CRC and aid future diapeutics investigations. The robustness of the present findings provides the opportunity to re-evaluate the genes associated with diseases and expand the gene-disease databases. The report also highlights LRI algorithms aided genes assessment to evaluate their contribution as a major factor with an etiological role in complex human disease conditions.

## Supplementary Information

Supplementary Information 1.

Supplementary Information 2.

## Data Availability

The meta-dataset analysed (E-MTAB-6698) during the current study are available in the [Arrayexpress] repository, [https://www.ebi.ac.uk/arrayexpress/experiments/E-MTAB-6698/]. All data generated during this study are included in this published article [and its supplementary information files].The in-house script for calculating the LRI is available at https://github.com/Vishwabaruah/GT_LRI.

## Abbreviations

LRI: Link Relevance Index
CRC: Colorectal cancer
GO: Gene Ontology
BP: Biological Processes
MF: Molecular Function
CC: Cellular component
KEGG: Kyoto Encyclopedia of Genes and Genomes
TCGA: The Cancer Genome Atlas
