# Supplementary material for: Game-theoretic link relevance indexing on genome-wide expression dataset identifies putative salient genes with potential etiological and diapeutics role in colorectal cancer
Source: Sci Rep. 2022 Aug 4;12:13409. doi: 10.1038/s41598-022-17266-0 (PMC9352798; doi:10.1038/s41598-022-17266-0)
Supplement: Supplementary file 1 — Supplementary Information 1. [file 41598_2022_17266_MOESM1_ESM.docx]

**Supplementary File**

Table S1: List of 126 salient genes with a positive LRI score along with gene's description.

| **Sl no** | **Input gene ID (AffySYMBOL)** | **LRI Score** | **Entrez ID** | **Synonyms** | **Type of gene** | **Chromosome** | **Map location** | **Full name from nomenclature authority** |
| --- | --- | --- | --- | --- | --- | --- | --- | --- |
| 1 | MIR143HG | 0.016045 | 728264 | CARMEN, MIR143HG | ncRNA | 5 | 5q32 | cardiac mesoderm enhancer-associated non-coding RNA |
| 2 | AMOTL1 | 0.016045 | 154810 | JEAP | protein-coding | 11 | 11q21 | angiomotin like 1 |
| 3 | ACTG2 | 0.015873 | 72 | ACT, ACTA3, ACTE, ACTL3, ACTSG, VSCM | protein-coding | 2 | 2p13.1 | actin gamma 2, smooth muscle |
| 4 | FILIP1 | 0.011054 | 27145 | FILIP | protein-coding | 6 | 6q14.1 | filamin A interacting protein 1 |
| 5 | ARHGEF17 | 0.011054 | 9828 | P164RHOGEF, RHOGEF17, TEM4, p164-RhoGEF | protein-coding | 11 | 11q13.4 | Rho guanine nucleotide exchange factor 17 |
| 6 | FAM219B | 0.011054 | 57184 | C15orf17 | protein-coding | 15 | 15q24.1-q24.2 | family with sequence similarity 219 member B |
| 7 | ITPKB | 0.00959 | 3707 | IP3-3KB, IP3K, IP3K-B, IP3KB, PIG37 | protein-coding | 1 | 1q42.12 | inositol-trisphosphate 3-kinase B |
| 8 | TOP2A | 0.00959 | 7153 | TOP2, TP2A | protein-coding | 17 | 17q21.2 | DNA topoisomerase II alpha |
| 9 | HAND1 | 0.00959 | 9421 | Hxt, Thing1, bHLHa27, eHand | protein-coding | 5 | 5q33.2 | heart and neural crest derivatives expressed 1 |
| 10 | SERINC2 | 0.00959 | 347735 | FKSG84, PRO0899, TDE2, TDE2L | protein-coding | 1 | 1p35.2 | serine incorporator 2 |
| 11 | TRAP1 | 0.009081 | 10131 | HSP 75, HSP75, HSP90L, TRAP-1 | protein-coding | 16 | 16p13.3 | TNF receptor associated protein 1 |
| 12 | CAMSAP1 | 0.009081 | 157922 |  | protein-coding | 9 | 9q34.3 | calmodulin regulated spectrin associated protein 1 |
| 13 | APOBR | 0.009081 | 55911 | APOB100R, APOB48R | protein-coding | 16 | 16p12.1 | apolipoprotein B receptor |
| 14 | PAG1 | 0.009081 | 55824 | CBP, PAG | protein-coding | 8 | 8q21.13 | phosphoprotein membrane anchor with glycosphingolipid microdomains 1 |
| 15 | MRPS9 | 0.009081 | 64965 | MRP-S9, RPMS9, S9mt | protein-coding | 2 | 2q12.1 | mitochondrial ribosomal protein S9 |
| 16 | KANK2 | 0.008564 | 25959 | ANKRD25, MXRA3, NPHS16, PPKWH, SIP | protein-coding | 19 | 19p13.2 | KN motif and ankyrin repeat domains 2 |
| 17 | EML1 | 0.008564 | 2009 | BH, ELP79, EMAP, EMAP-1, EMAPL | protein-coding | 14 | 14q32.2 | EMAP like 1 |
| 18 | SCGB2A1 | 0.008244 | 4246 | LPHC, LPNC, MGB2, UGB3 | protein-coding | 11 | 11q12.3 | secretoglobin family 2A member 1 |
| 19 | CFAP53 | 0.007937 | 220136 | CCDC11, HTX6 | protein-coding | 18 | 18q21.1 | cilia and flagella associated protein 53 |
| 20 | SLC26A7 | 0.007937 | 115111 | SUT2 | protein-coding | 8 | 8q21.3 | solute carrier family 26 member 7 |
| 21 | CDK8 | 0.007937 | 1024 | IDDHBA, K35 | protein-coding | 13 | 13q12.13 | cyclin dependent kinase 8 |
| 22 | CHRM3 | 0.007937 | 1131 | EGBRS, HM3, PBS | protein-coding | 1 | 1q43 | cholinergic receptor muscarinic 3 |
| 23 | PTBP2 | 0.007937 | 58155 | PTBLP, brPTB, nPTB | protein-coding | 1 | 1p21.3 | polypyrimidine tract binding protein 2 |
| 24 | LOC100129461 | 0.007937 | 100129461 |  | ncRNA | 6 | 6p25.1 | hypothetical protein LOC100129461 |
| 25 | LOC105374419 | 0.007937 | 105374419 |  | ncRNA | 4 | 4p14 |  |
| 26 | TTLL7-IT1 | 0.007937 | 100874314 |  | ncRNA | 1 | 1p31.1 | TTLL7 intronic transcript 1 |
| 27 | LOC400965 | 0.007937 | 400965 |  |  | 2 | 2p11.2 | hypothetical LOC400965 |
| 28 | CWF19L2 | 0.007937 | 143884 |  | protein-coding | 11 | 11q22.3 | CWF19 like cell cycle control factor 2 |
| 29 | OR5L2 | 0.007937 | 26338 | HSHTPCRX16, HTPCRX16, OR11-153 | protein-coding | 11 | 11q12.1 | olfactory receptor family 5 subfamily L member 2 |
| 30 | CECR9 | 0.007937 | 30847 |  | ncRNA | 22 | 22q11.2 | cat eye syndrome chromosome region, candidate 9 |
| 31 | VCL | 0.007937 | 7414 | CMD1W, CMH15, HEL114, MV, MVCL | protein-coding | 10 | 10q22.2 | vinculin |
| 32 | EBP | 0.007937 | 10682 | CDPX2, CHO2, CPX, CPXD, MEND | protein-coding | X | Xp11.23 | EBP cholestenol delta-isomerase |
| 33 | EPM2AIP1 | 0.007937 | 9852 |  | protein-coding | 3 | 3p22.2 | EPM2A interacting protein 1 |
| 34 | SLC25A12 | 0.007937 | 8604 | AGC1, ARALAR, EIEE39 | protein-coding | 2 | 2q31.1 | solute carrier family 25 member 12 |
| 35 | SORL1 | 0.007937 | 6653 | C11orf32, LR11, LRP9, SORLA, SorLA-1, gp250 | protein-coding | 11 | 11q24.1 | sortilin related receptor 1 |
| 36 | LMOD1 | 0.007937 | 25802 | 1D, 64kD, D1, SM-LMOD, SMLMOD | protein-coding | 1 | 1q32.1 | leiomodin 1 |
| 37 | ADARB1 | 0.007937 | 104 | ADAR2, DRABA2, DRADA2, NEDHYMS, RED1 | protein-coding | 21 | 21q22.3 | adenosine deaminase RNA specific B1 |
| 38 | RFC3 | 0.007937 | 5983 | RFC38 | protein-coding | 13 | 13q13.2 | replication factor C subunit 3 |
| 39 | FAAH | 0.007937 | 2166 | FAAH-1, PSAB | protein-coding | 1 | 1p33 | fatty acid amide hydrolase |
| 40 | CCDC94 | 0.007937 | 55702 | CCDC94 | protein-coding | 19 | 19p13.3 | YJU2 splicing factor homolog |
| 41 | NFE2L3 | 0.007937 | 9603 | NRF3 | protein-coding | 7 | 7p15.2 | nuclear factor, erythroid 2 like 3 |
| 42 | PLN | 0.007937 | 5350 | CMD1P, CMH18, PLB | protein-coding | 6 | 6q22.31 | phospholamban |
| 43 | ACTC1 | 0.007937 | 70 | ACTC, ASD5, CMD1R, CMH11, LVNC4 | protein-coding | 15 | 15q14 | actin alpha cardiac muscle 1 |
| 44 | MSX2 | 0.007937 | 4488 | CRS2, FPP, HOX8, MSH, PFM, PFM1 | protein-coding | 5 | 5q35.2 | msh homeobox 2 |
| 45 | RXRG | 0.007937 | 6258 | NR2B3, RXRC | protein-coding | 1 | 1q23.3 | retinoid X receptor gamma |
| 46 | PRKCE | 0.007937 | 5581 | PKCE, nPKC-epsilon | protein-coding | 2 | 2p21 | protein kinase C epsilon |
| 47 | RYR3 | 0.007937 | 6263 | RYR-3 | protein-coding | 15 | 15q13.3-q14 | ryanodine receptor 3 |
| 48 | GNAL | 0.007937 | 2774 | DYT25 | protein-coding | 18 | 18p11.21 | G protein subunit alpha L |
| 49 | MASP1 | 0.007937 | 5648 | 3MC1, CRARF, CRARF1, MAP1, MASP, MASP3, MAp44, PRSS5, RaRF | protein-coding | 3 | 3q27.3 | mannan binding lectin serine peptidase 1 |
| 50 | SMARCA2 | 0.007937 | 6595 | BAF190, BRM, NCBRS, SNF2, SNF2L2, SNF2LA, SWI2, Sth1p, hBRM, hSNF2a | protein-coding | 9 | 9p24.3 | SWI/SNF related, matrix associated, actin dependent regulator of chromatin, subfamily a, member 2 |
| 51 | DKK4 | 0.007937 | 27121 | DKK-4 | protein-coding | 8 | 8p11.21 | dickkopf WNT signaling pathway inhibitor 4 |
| 52 | SI | 0.007937 | 6476 |  | protein-coding | 3 | 3q26.1 | sucrase-isomaltase |
| 53 | FRMPD1 | 0.007937 | 22844 | FRMD2 | protein-coding | 9 | 9p13.2 | FERM and PDZ domain containing 1 |
| 54 | SLC16A7 | 0.007937 | 9194 | MCT2 | protein-coding | 12 | 12q14.1 | solute carrier family 16 member 7 |
| 55 | CASQ2 | 0.007937 | 845 | PDIB2 | protein-coding | 1 | 1p13.1 | calsequestrin 2 |
| 56 | BTC | 0.007937 | 685 |  | protein-coding | 4 | 4q13.3 | betacellulin |
| 57 | TLX2 | 0.007937 | 3196 | HOX11L1, NCX | protein-coding | 2 | 2p13.1 | T cell leukemia homeobox 2 |
| 58 | LILRA2 | 0.007937 | 11027 | CD85H, ILT1, LIR-7, LIR7 | protein-coding | 19 | 19q13.42 | leukocyte immunoglobulin like receptor A2 |
| 59 | CTGF | 0.007937 | 1490 | CTGF, HCS24, IGFBP8, NOV2 | protein-coding | 6 | 6q23.2 | cellular communication network factor 2 |
| 60 | EXTL3 | 0.007937 | 2137 | BOTV, EXTL1L, EXTR1, ISDNA, REGR, RPR | protein-coding | 8 | 8p21.1 | exostosin like glycosyltransferase 3 |
| 61 | BAG2 | 0.007937 | 9532 | BAG-2, dJ417I1.2 | protein-coding | 6 | 6p12.1 | BAG cochaperone 2 |
| 62 | OCLN | 0.007937 | 100506658 | BLCPMG, PPP1R115, PTORCH1 | protein-coding | 5 | 5q13.2 | occludin |
| 63 | SERPINE2 | 0.007937 | 5270 | GDN, GDNPF, PI-7, PI7, PN-1, PN1, PNI | protein-coding | 2 | 2q36.1 | serpin family E member 2 |
| 64 | SAMD4A | 0.007937 | 23034 | SAMD4, SMAUG, SMAUG1, SMG, SMGA | protein-coding | 14 | 14q22.2 | sterile alpha motif domain containing 4A |
| 65 | MFSD9 | 0.007937 | 84804 |  | protein-coding | 2 | 2q12.1 | major facilitator superfamily domain containing 9 |
| 66 | IFITM1 | 0.007937 | 8519 | 9-27, CD225, DSPA2a, IFI17, LEU13 | protein-coding | 11 | 11p15.5 | interferon induced transmembrane protein 1 |
| 67 | TSPAN2 | 0.007937 | 10100 | NET3, TSN2, TSPAN-2 | protein-coding | 1 | 1p13.2 | tetraspanin 2 |
| 68 | LPXN | 0.007937 | 9404 | LDPL | protein-coding | 11 | 11q12.1 | leupaxin |
| 69 | CCL2 | 0.007937 | 6347 | GDCF-2, HC11, HSMCR30, MCAF, MCP-1, MCP1, SCYA2, SMC-CF | protein-coding | 17 | 17q12 | C-C motif chemokine ligand 2 |
| 70 | MT1M | 0.007937 | 4499 | MT-1M, MT-IM, MT1, MT1K | protein-coding | 16 | 16q13 | metallothionein 1M |
| 71 | FXYD6 | 0.007937 | 53826 |  | protein-coding | 11 | 11q23.3 | FXYD domain containing ion transport regulator 6 |
| 72 | NOL8 | 0.007937 | 55035 | C9orf34, NOP132, bA62C3.3, bA62C3.4 | protein-coding | 9 | 9q22.31 | nucleolar protein 8 |
| 73 | MSL2 | 0.007937 | 55167 | MSL-2, MSL2L1, RNF184 | protein-coding | 3 | 3q22.3 | MSL complex subunit 2 |
| 74 | ZFAND1 | 0.007937 | 79752 |  | protein-coding | 8 | 8q21.13 | zinc finger AN1-type containing 1 |
| 75 | LIPG | 0.007937 | 9388 | EDL, EL, PRO719 | protein-coding | 18 | 18q21.1 | lipase G, endothelial type |
| 76 | PRDM10 | 0.007937 | 56980 | PFM7, TRIS | protein-coding | 11 | 11q24.3 | PR/SET domain 10 |
| 77 | SLC2A9 | 0.007937 | 56606 | GLUT9, GLUTX, UAQTL2, URATv1 | protein-coding | 4 | 4p16.1 | solute carrier family 2 member 9 |
| 78 | PDZRN4 | 0.007937 | 29951 | LNX4, SAMCAP3L | protein-coding | 12 | 12q12 | PDZ domain containing ring finger 4 |
| 79 | C19orf24 | 0.007937 | 55009 | C19orf24 | protein-coding | 19 | 19p13.3 | family with sequence similarity 174 member C |
| 80 | R3HDM4 | 0.007937 | 91300 | C19orf22 | protein-coding | 19 | 19p13.3 | R3H domain containing 4 |
| 81 | TIMMDC1 | 0.007937 | 51300 | C3orf1, MC1DN31 | protein-coding | 3 | 3q13.33 | translocase of inner mitochondrial membrane domain containing 1 |
| 82 | PPAPDC1B | 0.007937 | 84513 | DPPL1, HTPAP, PPAPDC1B | protein-coding | 8 | 8p11.23 | phospholipid phosphatase 5 |
| 83 | KATNAL1 | 0.007937 | 84056 |  | protein-coding | 13 | 13q12.3 | katanin catalytic subunit A1 like 1 |
| 84 | INTS2 | 0.007937 | 57508 | INT2, KIAA1287 | protein-coding | 17 | 17q23.2 | integrator complex subunit 2 |
| 85 | ANGPTL1 | 0.007937 | 9068 | ANG3, ANGPT3, ARP1, AngY, UNQ162, dJ595C2.2 | protein-coding | 1 | 1q25.2 | angiopoietin like 1 |
| 86 | METRNL | 0.007937 | 284207 |  | protein-coding | 17 | 17q25.3 | meteorin like, glial cell differentiation regulator |
| 87 | TSTD1 | 0.007937 | 100131187 | KAT, TST | protein-coding | 1 | 1q23.3 | thiosulfate sulfurtransferase like domain containing 1 |
| 88 | LRRN1 | 0.007937 | 57633 | FIGLER3, NLRR-1 | protein-coding | 3 | 3p26.2 | leucine rich repeat neuronal 1 |
| 89 | ALDH16A1 | 0.007937 | 126133 |  | protein-coding | 19 | 19q13.33 | aldehyde dehydrogenase 16 family member A1 |
| 90 | KIAA1804 | 0.007937 | 84451 | MLK4,RP5-862P8.2,dJ862P8.3, | protein-coding | 1 | 1q42 | mixed lineage kinase 4 |
| 91 | MYOCD | 0.007937 | 93649 | MGBL, MYCD | protein-coding | 17 | 17p12 | myocardin |
| 92 | WIPF3 | 0.007937 | 644150 | CR16 | protein-coding | 7 | 7p14.3 | WAS/WASL interacting protein family member 3 |
| 93 | MORN5 | 0.007937 | 254956 | C9orf113, C9orf18 | protein-coding | 9 | 9q33.2 | MORN repeat containing 5 |
| 94 | NUMB | 0.007937 | 8650 | C14orf41, S171, c14_5527 | protein-coding | 14 | 14q24.2-q24.3 | NUMB endocytic adaptor protein |
| 95 | LINC01224 | 0.007937 | 104472717 |  | ncRNA | 19 | 19p12 | long intergenic non-protein coding RNA 1224 |
| 96 | ITIH6 | 0.007937 | 347365 | ITIH5L, UNQ6369, dJ14O9.1 | protein-coding | X | Xp11.22 | inter-alpha-trypsin inhibitor heavy chain family member 6 |
| 97 | CMTM8 | 0.007937 | 152189 | CKLFSF8, CKLFSF8-V2 | protein-coding | 3 | 3p22.3 | CKLF like MARVEL transmembrane domain containing 8 |
| 98 | LINC00202-2 | 0.007937 | 731789 | LINC00202-2 | ncRNA | 10 | 10p12.1 | family with sequence similarity 238 member B |
| 99 | ACSM2A | 0.007937 | 123876 | A-923A4.1, ACSM2 | protein-coding | 16 | 16p12.3 | acyl-CoA synthetase medium chain family member 2A |
| 100 | SLC9A2 | 0.007527 | 6549 | NHE2 | protein-coding | 2 | 2q12.1 | solute carrier family 9 member A2 |
| 101 | SGCE | 0.007275 | 8910 | DYT11, ESG, epsilon-SG | protein-coding | 7 | 7q21.3 | sarcoglycan epsilon |
| 102 | FOXF1 | 0.007275 | 2294 | ACDMPV, FKHL5, FREAC1 | protein-coding | 16 | 16q24.1 | forkhead box F1 |
| 103 | MEIS2 | 0.007275 | 4212 | CPCMR, HsT18361, MRG1 | protein-coding | 15 | 15q14 | Meis homeobox 2 |
| 104 | TMEM125 | 0.007275 | 128218 |  | protein-coding | 1 | 1p34.2 | transmembrane protein 125 |
| 105 | TPM1 | 0.007146 | 7168 | C15orf13, CMD1Y, CMH3, HEL-S-265, HTM-alpha, LVNC9, TMSA | protein-coding | 15 | 15q22.2 | tropomyosin 1 |
| 106 | AHNAK | 0.007146 | 79026 | AHNAKRS, PM227 | protein-coding | 11 | 11q12.3 | AHNAK nucleoprotein |
| 107 | WFDC1 | 0.007146 | 58189 | PS20 | protein-coding | 16 | 16q24.1 | WAP four-disulfide core domain 1 |
| 108 | MFAP3L | 0.006889 | 9848 | NYD-sp9 | protein-coding | 4 | 4q33 | microfibril associated protein 3 like |
| 109 | TOX2 | 0.006889 | 84969 | C20orf100, GCX-1, GCX1, dJ1108D11.2, dJ495O3.1 | protein-coding | 20 | 20q13.12 | TOX high mobility group box family member 2 |
| 110 | ITGA5 | 0.006661 | 3678 | CD49e, FNRA, VLA-5, VLA5A | protein-coding | 12 | 12q13.13 | integrin subunit alpha 5 |
| 111 | SLC25A4 | 0.006661 | 291 | AAC1, ANT, ANT 1, ANT1, MTDPS12, MTDPS12A, PEO2, PEO3, PEOA2, T1 | protein-coding | 4 | 4q35.1 | solute carrier family 25 member 4 |
| 112 | SLCO4C1 | 0.006393 | 353189 | OATP-H, OATP-M1, OATP4C1, OATPX, PRO2176, SLC21A20 | protein-coding | 5 | 5q21.1 | solute carrier organic anion transporter family member 4C1 |
| 113 | GPM6B | 0.005952 | 2824 | M6B | protein-coding | X | Xp22.2 | glycoprotein M6B |
| 114 | SECTM1 | 0.005952 | 6398 | K12, SECTM | protein-coding | 17 | 17q25.3 | secreted and transmembrane 1 |
| 115 | POPDC2 | 0.005952 | 64091 | POP2 | protein-coding | 3 | 3q13.33 | popeye domain containing 2 |
| 116 | MCAM | 0.005669 | 4162 | CD146, HEMCAM, METCAM, MUC18, MelCAM | protein-coding | 11 | 11q23.3 | melanoma cell adhesion molecule |
| 117 | RGS2 | 0.005669 | 5997 | G0S8 | protein-coding | 1 | 1q31.2 | regulator of G protein signaling 2 |
| 118 | ADAMTS9-AS1 | 0.005669 | 101929335 |  | ncRNA | 3 | 3p14.1 | ADAMTS9 antisense RNA 1 |
| 119 | MYL9 | 0.005291 | 10398 | LC20, MLC-2C, MLC2, MRLC1, MYRL2 | protein-coding | 20 | 20q11.23 | myosin light chain 9 |
| 120 | CNN1 | 0.005291 | 1264 | HEL-S-14, SMCC, Sm-Calp | protein-coding | 19 | 19p13.2 | calponin 1 |
| 121 | MAB21L2 | 0.005291 | 10586 | MCOPS14, MCSKS14 | protein-coding | 4 | 4q31.3 | mab-21 like 2 |
| 122 | PPP1R14A | 0.005257 | 94274 | CPI-17, CPI17, PPP1INL | protein-coding | 19 | 19q13.2 | protein phosphatase 1 regulatory inhibitor subunit 14A |
| 123 | RNF150 | 0.005257 | 57484 |  | protein-coding | 4 | 4q31.21 | ring finger protein 150 |
| 124 | FLNA | 0.00469 | 2316 | ABP-280, ABPX, CSBS, CVD1, FGS2, FLN, FLN-A, FLN1, FMD, MNS, NHBP, OPD, OPD1, OPD2, XLVD, XMVD | protein-coding | X | Xq28 | filamin A |
| 125 | FOXF2 | 0.00469 | 2295 | FKHL6, FREAC-2, FREAC2 | protein-coding | 6 | 6p25.3 | forkhead box F2 |
| 126 | UBXN10-AS1 | 0.00469 | 391013 | UBXN10-AS1 | protein-coding | 1 | 1p36.12 | phospholipase A2 group IIC |

Table S2: List of overrepresented Biological Process (BP) terms associated with salient genes. Ontology Source: GO_BiologicalProcess-EBI-UniProt-GOA-ACAP-ARAP_08.05.2020_00h00

| **GO ID** | **GO Term** | **Term P Value** | **Term P Value Corrected with Benjamini-Hochberg** | **Group P Value** | **Group P Value Corrected with Benjamini-Hochberg** | **GO Levels** | **GO Groups** | **% Associated Genes** | **# Genes** | **Associated Genes Found** |
| --- | --- | --- | --- | --- | --- | --- | --- | --- | --- | --- |
| GO:0005219 | ryanodine-sensitive calcium-release channel activity | 0.000922054 | 0.001267825 | 0.000922054 | 0.001290876 | [6, 7, 8, 9, 10, 11, 12, 13, 14, 15, 16] | Group0 | 9.375 | 3 | [CASQ2, PLN, RYR3] |
| GO:0044319 | wound healing, spreading of cells | 0.001102758 | 0.001347815 | 0.001102758 | 0.001286551 | [4, 5, 6, 8, 9] | Group1 | 8.823529243 | 3 | [FLNA, ITGA5, MSX2] |
| GO:0060415 | muscle tissue morphogenesis | 0.001250227 | 0.00137525 | 0.001250227 | 0.001250227 | [4, 5, 6, 7, 8] | Group2 | 5.128205299 | 4 | [ACTC1, ADARB1, HAND1, TPM1] |
| GO:0070527 | platelet aggregation | 0.000593879 | 0.000933238 | 0.000593879 | 0.001039288 | [4, 5, 7] | Group3 | 6.25 | 4 | [FLNA, MYL9, SERPINE2, VCL] |
| GO:0072132 | mesenchyme morphogenesis | 0.00033226 | 0.000730972 | 0.00033226 | 0.000775274 | [4, 5, 6, 7] | Group4 | 7.272727489 | 4 | [ACTC1, ACTG2, FOXF1, MSX2] |
| GO:0006937 | regulation of muscle contraction | 8.56166E-08 | 9.41782E-07 | 8.56166E-08 | 5.99316E-07 | [5, 6] | Group5 | 5.8139534 | 10 | [CASQ2, CCN2, CHRM3, CNN1, FLNA, MYL9, MYOCD, PLN, RGS2, TPM1] |
| GO:0055117 | regulation of cardiac muscle contraction | 0.000188219 | 0.001035203 | 8.56166E-08 | 5.99316E-07 | [6, 7, 8] | Group5 | 5.681818008 | 5 | [CASQ2, CCN2, FLNA, PLN, RGS2] |
| GO:1903524 | positive regulation of blood circulation | 0.001505142 | 0.001505142 | 0.000200196 | 0.000700686 | [3, 4, 5, 6] | Group6 | 4.878048897 | 4 | [CCN2, CHRM3, RGS2, TPM1] |
| GO:0045933 | positive regulation of muscle contraction | 0.000247983 | 0.000681954 | 0.000200196 | 0.000700686 | [3, 4, 5, 6, 7] | Group6 | 7.843137264 | 4 | [CCN2, CHRM3, MYOCD, RGS2] |
| GO:1904706 | negative regulation of vascular smooth muscle cell proliferation | 0.000190659 | 0.000699084 | 0.000200196 | 0.000700686 | [5, 6, 7] | Group6 | 15.78947353 | 3 | [CNN1, MYOCD, TPM1] |
| GO:0006940 | regulation of smooth muscle contraction | 0.000559328 | 0.001025434 | 0.000200196 | 0.000700686 | [6, 7] | Group6 | 6.349206448 | 4 | [CHRM3, CNN1, MYOCD, RGS2] |

Table S3: List of overrepresented Molecular Function (MF) term associated with salient genes. Ontology Source: GO_MolecularFunction-EBI-UniProt-GOA-ACAP-ARAP_08.05.2020_00h00

| **GO ID** | **GO Term** | **Term P Value** | **Term P Value Corrected with Benjamini- Hochberg** | **Group P Value** | **Group P Value Corrected with Benjamini- Hochberg** | **GO Levels** | **GO Groups** | **% Associated Genes** | **# Genes** | **Associated Genes Found** |
| --- | --- | --- | --- | --- | --- | --- | --- | --- | --- | --- |
| GO:0032412 | regulation of ion transmembrane transporter activity | 0.001925 | 0.001925 | 0.001925 | 0.001925 | [4] | Group0 | 2.439024 | 7 | [AHNAK, CASQ2, CCL2, CHRM3, FXYD6, PLN, PRKCE] |

Table S4: List of overrepresented Cellular component (CC) terms associated with salient genes. Ontology Source: GO_CellularComponent-EBI-UniProt-GOA-ACAP-ARAP_08.05.2020_00h00

| **GO ID** | **GO Term** | **Term P Value** | **Term P Value Corrected with Benjamini-**  **Hochberg** | **Group P Value** | **Group P Value Corrected with Benjamini-**  **Hochberg** | **GO Levels** | **GO Groups** | **% Associated Genes** | **# Genes** | **Associated Genes Found** |
| --- | --- | --- | --- | --- | --- | --- | --- | --- | --- | --- |
| GO:0033017 | sarcoplasmic reticulum membrane | 0.002005 | 0.002673 | 0.002005 | 0.002005 | [5, 6, 7] | Group0 | 7.142857 | 3 | [CASQ2, PLN, RYR3] |
| GO:0030016 | myofibril | 1.47E-05 | 5.9E-05 | 1.47E-05 | 2.95E-05 | [4, 5, 6] | Group1 | 3.719008 | 9 | [ACTC1, AHNAK, CASQ2, FLNA, LMOD1, MYL9, RYR3, TPM1, VCL] |
| GO:0030017 | sarcomere | 0.000354 | 0.000707 | 1.47E-05 | 2.95E-05 | [2, 5, 6, 7] | Group1 | 3.181818 | 7 | [ACTC1, CASQ2, FLNA, LMOD1, MYL9, RYR3, TPM1] |
| GO:0031674 | I band | 0.002312 | 0.002312 | 1.47E-05 | 2.95E-05 | [2, 3, 6, 7, 8] | Group1 | 3.246753 | 5 | [ACTC1, CASQ2, FLNA, MYL9, RYR3] |

Table S5: List of overrepresented KEGG pathways terms associated with salient genes. Ontology Source: KEGG_08.05.2020 and GO_BiologicalProcess-EBI-UniProt-GOA-ACAP-ARAP_08.05.2020_00h00

| **Sl no** | **GO ID** | **GO Term** | **Term P Value** | **Term P Value Corrected with Benjamini-Hochberg** | **Group P Value** | **Group P Value Corrected with Benjamini-Hochberg** | **GO Levels** | **GO Groups** | **% Associated Genes** | **# Genes** | **Associated Genes Found** |
| --- | --- | --- | --- | --- | --- | --- | --- | --- | --- | --- | --- |
| 1 | GO:0044319 | wound healing, spreading of cells | 0.000548 | 0.001508 | 0.000548 | 0.000987 | [4, 5, 6, 8, 9] | Group0 | 8.823529 | 3 | [FLNA, ITGA5, MSX2] |
| 2 | GO:0046464 | acylglycerol catabolic process | 0.018495 | 0.018495 | 0.018495 | 0.018495 | [6, 7] | Group1 | 4.081633 | 2 | [FAAH, SORL1] |
| 3 | GO:0051797 | regulation of hair follicle development | 0.004197 | 0.006295 | 0.004197 | 0.004721 | [4, 5, 6, 7, 8, 9] | Group2 | 8.695652 | 2 | [DKK4, MSX2] |
| 4 | GO:0070527 | platelet aggregation | 0.000298 | 0.001227 | 0.000298 | 0.000669 | [4, 5, 7] | Group3 | 6.25 | 4 | [FLNA, MYL9, SERPINE2, VCL] |
| 5 | GO:0072132 | mesenchyme morphogenesis | 0.000165 | 0.001088 | 0.000165 | 0.000494 | [4, 5, 6, 7] | Group4 | 7.272727 | 4 | [ACTC1, ACTG2, FOXF1, MSX2] |
| 6 | GO:0010676 | positive regulation of cellular carbohydrate metabolic process | 0.003816 | 0.006296 | 0.003816 | 0.004906 | [4, 5, 6, 7] | Group5 | 4.545455 | 3 | [EPM2AIP1, PRKCE, SLC25A12] |
| 7 | GO:0005347 | ATP transmembrane transporter activity | 0.006207 | 0.008193 | 0.002506 | 0.003759 | [6, 7, 8, 9, 10, 11] | Group6 | 7.142857 | 2 | [SLC25A12, SLC25A4] |
| 8 | GO:0015301 | anion:anion antiporter activity | 0.009101 | 0.010726 | 0.002506 | 0.003759 | [7, 8, 9, 10] | Group6 | 5.882353 | 2 | [SLC25A4, SLC26A7] |
| 9 | GO:1903524 | positive regulation of blood circulation | 0.000769 | 0.001693 | 4.8E-07 | 2.16E-06 | [3, 4, 5, 6] | Group7 | 4.878049 | 4 | [CCN2, CHRM3, RGS2, TPM1] |
| 10 | GO:0045933 | positive regulation of muscle contraction | 0.000122 | 0.00101 | 4.8E-07 | 2.16E-06 | [3, 4, 5, 6, 7] | Group7 | 7.843137 | 4 | [CCN2, CHRM3, MYOCD, RGS2] |
| 11 | GO:0006937 | regulation of muscle contraction | 3.95E-08 | 1.3E-06 | 4.8E-07 | 2.16E-06 | [5, 6] | Group7 | 5.813953 | 10 | [CASQ2, CCN2, CHRM3, CNN1, FLNA, MYL9, MYOCD, PLN, RGS2, TPM1] |
| 12 | GO:0014909 | smooth muscle cell migration | 0.005482 | 0.007537 | 4.8E-07 | 2.16E-06 | [5, 6] | Group7 | 4 | 3 | [MYOCD, SORL1, TPM1] |
| 13 | GO:0030049 | muscle filament sliding | 0.011898 | 0.013539 | 4.8E-07 | 2.16E-06 | [5, 6] | Group7 | 5.128205 | 2 | [ACTC1, TPM1] |
| 14 | GO:0014910 | regulation of smooth muscle cell migration | 0.003983 | 0.006258 | 4.8E-07 | 2.16E-06 | [5, 6, 7] | Group7 | 4.477612 | 3 | [MYOCD, SORL1, TPM1] |
| 15 | GO:0045987 | positive regulation of smooth muscle contraction | 0.008079 | 0.010254 | 4.8E-07 | 2.16E-06 | [4, 5, 6, 7, 8] | Group7 | 6.25 | 2 | [CHRM3, MYOCD] |
| 16 | GO:1904706 | negative regulation of vascular smooth muscle cell proliferation | 9.28E-05 | 0.00153 | 4.8E-07 | 2.16E-06 | [5, 6, 7] | Group7 | 15.78947 | 3 | [CNN1, MYOCD, TPM1] |
| 17 | GO:0006940 | regulation of smooth muscle contraction | 0.00028 | 0.00132 | 4.8E-07 | 2.16E-06 | [6, 7] | Group7 | 6.349206 | 4 | [CHRM3, CNN1, MYOCD, RGS2] |
| 18 | GO:0045214 | sarcomere organization | 0.01638 | 0.016892 | 4.8E-07 | 2.16E-06 | [5, 6, 7, 8, 9] | Group7 | 4.347826 | 2 | [CASQ2, TPM1] |
| 19 | GO:0055117 | regulation of cardiac muscle contraction | 9.37E-05 | 0.001031 | 4.8E-07 | 2.16E-06 | [6, 7, 8] | Group7 | 5.681818 | 5 | [CASQ2, CCN2, FLNA, PLN, RGS2] |
| 20 | GO:1904753 | negative regulation of vascular associated smooth muscle cell migration | 0.000257 | 0.001415 | 4.8E-07 | 2.16E-06 | [6, 7, 8, 9] | Group7 | 33.33333 | 2 | [MYOCD, TPM1] |
| 21 | KEGG:05414 | Dilated cardiomyopathy (DCM) | 0.001392 | 0.002701 | 2.06E-11 | 1.86E-10 | [-1] | Group8 | 4.166667 | 4 | [ACTC1, ITGA5, PLN, TPM1] |
| 22 | GO:0005219 | ryanodine-sensitive calcium-release channel activity | 0.000457 | 0.001509 | 2.06E-11 | 1.86E-10 | [6, 7, 8, 9, 10, 11, 12, 13, 14, 15, 16] | Group8 | 9.375 | 3 | [CASQ2, PLN, RYR3] |
| 23 | GO:0014808 | release of sequestered calcium ion into cytosol by sarcoplasmic reticulum | 0.008583 | 0.01049 | 2.06E-11 | 1.86E-10 | [6, 7, 8, 9, 10, 12, 13, 14, 15, 16] | Group8 | 6.060606 | 2 | [CASQ2, PLN] |
| 24 | GO:0010881 | regulation of cardiac muscle contraction by regulation of the release of sequestered calcium ion | 0.003497 | 0.006074 | 2.06E-11 | 1.86E-10 | [6, 7, 8, 9, 10, 11, 12, 13, 14, 15, 16, 17, 18] | Group8 | 9.523809 | 2 | [CASQ2, PLN] |
| 25 | GO:0055119 | relaxation of cardiac muscle | 0.003171 | 0.005813 | 2.06E-11 | 1.86E-10 | [5] | Group8 | 10 | 2 | [PLN, RGS2] |
| 26 | GO:0006937 | regulation of muscle contraction | 3.95E-08 | 1.3E-06 | 2.06E-11 | 1.86E-10 | [5, 6] | Group8 | 5.813953 | 10 | [CASQ2, CCN2, CHRM3, CNN1, FLNA, MYL9, MYOCD, PLN, RGS2, TPM1] |
| 27 | GO:0030049 | muscle filament sliding | 0.011898 | 0.013539 | 2.06E-11 | 1.86E-10 | [5, 6] | Group8 | 5.128205 | 2 | [ACTC1, TPM1] |
| 28 | GO:0051581 | negative regulation of neurotransmitter uptake | 0.000478 | 0.001435 | 2.06E-11 | 1.86E-10 | [4, 5, 6, 7] | Group8 | 25 | 2 | [GPM6B, RGS2] |
| 29 | GO:0030239 | myofibril assembly | 0.000398 | 0.001459 | 2.06E-11 | 1.86E-10 | [4, 5, 6, 7, 8] | Group8 | 5.797101 | 4 | [ACTC1, CASQ2, LMOD1, TPM1] |
| 30 | GO:0060415 | muscle tissue morphogenesis | 0.000636 | 0.001615 | 2.06E-11 | 1.86E-10 | [4, 5, 6, 7, 8] | Group8 | 5.128205 | 4 | [ACTC1, ADARB1, HAND1, TPM1] |
| 31 | GO:0071313 | cellular response to caffeine | 0.000766 | 0.001805 | 2.06E-11 | 1.86E-10 | [5, 6, 7] | Group8 | 20 | 2 | [CASQ2, RYR3] |
| 32 | GO:0003208 | cardiac ventricle morphogenesis | 0.005278 | 0.007573 | 2.06E-11 | 1.86E-10 | [4, 5, 6, 7, 8, 9] | Group8 | 4.054054 | 3 | [FOXF1, HAND1, TPM1] |
| 33 | GO:1904063 | negative regulation of cation transmembrane transport | 0.001236 | 0.002549 | 2.06E-11 | 1.86E-10 | [5, 6, 7, 8] | Group8 | 4.301075 | 4 | [CASQ2, PLN, PRKCE, RGS2] |
| 34 | GO:0045214 | sarcomere organization | 0.01638 | 0.016892 | 2.06E-11 | 1.86E-10 | [5, 6, 7, 8, 9] | Group8 | 4.347826 | 2 | [CASQ2, TPM1] |
| 35 | GO:0055117 | regulation of cardiac muscle contraction | 9.37E-05 | 0.001031 | 2.06E-11 | 1.86E-10 | [6, 7, 8] | Group8 | 5.681818 | 5 | [CASQ2, CCN2, FLNA, PLN, RGS2] |
| 36 | GO:0086004 | regulation of cardiac muscle cell contraction | 0.01374 | 0.015113 | 2.06E-11 | 1.86E-10 | [5, 6, 7, 8, 9] | Group8 | 4.761905 | 2 | [FLNA, PLN] |
| 37 | GO:1904753 | negative regulation of vascular associated smooth muscle cell migration | 0.000257 | 0.001415 | 2.06E-11 | 1.86E-10 | [6, 7, 8, 9] | Group8 | 33.33333 | 2 | [MYOCD, TPM1] |
| 38 | GO:1901020 | negative regulation of calcium ion transmembrane transporter activity | 0.01438 | 0.015308 | 2.06E-11 | 1.86E-10 | [5, 6, 7, 8, 9, 10, 11, 12] | Group8 | 4.651163 | 2 | [CASQ2, PLN] |

Table S6: List of overrepresented REACTOME pathways terms associated with salient genes. Ontology Source: REACTOME_Pathways_08.05.2020

| **GO ID** | **GO Term** | **Term P Value** | **Term P Value Corrected with Benjamini-Hochberg** | **Group P Value** | **Group P Value Corrected with Benjamini-Hochberg** | **GO Levels** | **GO Groups** | **% Associated Genes** | **# Genes** | **Associated Genes Found** |
| --- | --- | --- | --- | --- | --- | --- | --- | --- | --- | --- |
| R-HSA:397014 | Muscle contraction | 1.89E-07 | 5.67E-07 | 1.89E-07 | 5.67E-07 | [-1] | Group0 | 4.830918 | 10 | [ACTC1, ACTG2, CASQ2, FXYD6, LMOD1, MYL9, PLN, RYR3, TPM1, VCL] |
| R-HSA:445355 | Smooth Muscle Contraction | 1.84E-06 | 2.77E-06 | 1.84E-06 | 2.77E-06 | [-1] | Group1 | 12.5 | 5 | [ACTG2, LMOD1, MYL9, TPM1, VCL] |
| R-HSA:5578775 | Ion homeostasis | 0.000176 | 0.000176 | 0.000176 | 0.000176 | [-1] | Group2 | 7.142857 | 4 | [CASQ2, FXYD6, PLN, RYR3] |

Table S7: List of overrepresented Reactome reactions terms associated with salient genes. Ontology Source: REACTOME_Reactions_08.05.2020

| **GO ID** | **GO Term** | **Term P Value** | **Term P Value Corrected with Benjamini-Hochberg** | **Group P Value** | **Group P Value Corrected with Benjamini-Hochberg** | **GO Levels** | **GO Groups** | **% Associated Genes** | **#**  **Genes** | **Associated Genes Found** |
| --- | --- | --- | --- | --- | --- | --- | --- | --- | --- | --- |
| R-HSA:445699 | ATP Hydrolysis By Myosin | 1.4E-07 | 1.4E-07 | 1.4E-07 | 1.4E-07 | [-1] | Group0 | 20 | 5 | [ACTG2, LMOD1, MYL9, TPM1, VCL] |
| R-HSA:445700 | Myosin Binds ATP | 1.4E-07 | 1.4E-07 | 1.4E-07 | 1.4E-07 | [-1] | Group0 | 20 | 5 | [ACTG2, LMOD1, MYL9, TPM1, VCL] |
| R-HSA:445704 | Calcium Binds Caldesmon | 1.4E-07 | 1.4E-07 | 1.4E-07 | 1.4E-07 | [-1] | Group0 | 20 | 5 | [ACTG2, LMOD1, MYL9, TPM1, VCL] |
| R-HSA:445705 | Release Of ADP From Myosin | 1.4E-07 | 1.4E-07 | 1.4E-07 | 1.4E-07 | [-1] | Group0 | 20 | 5 | [ACTG2, LMOD1, MYL9, TPM1, VCL] |

Table S8: List of significantly enriched clusters in protein-protein interaction (PPI) network found using STRING database along with associated salient genes.

| **Term ID** | **Term description** | **Observed gene count** | **Background gene count** | **Strength** | **False discovery rate** | **Matching proteins in the network (labels)** | **Matching proteins in the network (IDs)** |
| --- | --- | --- | --- | --- | --- | --- | --- |
| CL:1326 | mixed, incl. Muscle protein, and myofibril assembly | 10 | 131 | 1.11 | 5.09E-06 | CNN1,CASQ2,MYL9,ACTC1,PLN,TPM1,LMOD1,RYR3,ACTG2,MYOCD | 9606.ENSP00000252456,9606.ENSP00000261448,9606.ENSP00000279022,9606.ENSP00000290378,9606.ENSP00000350132,9606.ENSP00000351022,9606.ENSP00000356257,9606.ENSP00000373884,9606.ENSP00000386857,9606.ENSP00000401678 |
| CL:1328 | Muscle protein, and myofibril assembly | 8 | 104 | 1.11 | 7.51E-05 | CASQ2,MYL9,ACTC1,PLN,TPM1,LMOD1,RYR3,ACTG2 | 9606.ENSP00000261448,9606.ENSP00000279022,9606.ENSP00000290378,9606.ENSP00000350132,9606.ENSP00000351022,9606.ENSP00000356257,9606.ENSP00000373884,9606.ENSP00000386857 |
| CL:1331 | Muscle protein, and striated muscle thin filament | 5 | 65 | 1.11 | 0.0067 | MYL9,ACTC1,TPM1,LMOD1,ACTG2 | 9606.ENSP00000279022,9606.ENSP00000290378,9606.ENSP00000351022,9606.ENSP00000356257,9606.ENSP00000386857 |
| CL:1499 | sarcoplasmic reticulum membrane | 3 | 12 | 1.62 | 0.0079 | CASQ2,PLN,RYR3 | 9606.ENSP00000261448,9606.ENSP00000350132,9606.ENSP00000373884 |
| CL:1334 | RHO GTPases Activate ROCKs, and Smooth Muscle Contraction | 3 | 22 | 1.36 | 0.0241 | MYL9,LMOD1,ACTG2 | 9606.ENSP00000279022,9606.ENSP00000356257,9606.ENSP00000386857 |
| CL:25740 | mostly uncharacterized, incl. zonula adherens maintenance, and MyoD family inhibitor | 4 | 59 | 1.06 | 0.0267 | ALDH16A1,R3HDM4,C19orf24,ACSM2A | 9606.ENSP00000293350,9606.ENSP00000355385,9606.ENSP00000386557,9606.ENSP00000459451 |
| CL:25786 | mixed, incl. Midnolin, and R3H-associated N-terminal domain | 2 | 5 | 1.83 | 0.0327 | R3HDM4,C19orf24 | 9606.ENSP00000355385,9606.ENSP00000386557 |
| CL:1449 | myosin heavy chain binding, and actomyosin, actin portion | 2 | 6 | 1.75 | 0.0395 | ACTC1,TPM1 | 9606.ENSP00000290378,9606.ENSP00000351022 |
| CL:1577 | mixed, incl. negative regulation of amyloid-beta clearance, and Smoothelin | 2 | 6 | 1.75 | 0.0395 | CNN1,MYOCD | 9606.ENSP00000252456,9606.ENSP00000401678 |
| CL:6451 | mixed, incl. ectodermal digestive tract morphogenesis, and Tubulin polymerisation-promoting protein family member 1 | 2 | 8 | 1.62 | 0.0495 | FOXF2,FOXF1 | 9606.ENSP00000259806,9606.ENSP00000262426 |

#NB: Use following guidelines to understand the data.

1. Observed gene count indicates how many proteins in the network are annotated with a particular term. The second number (Background gene count) indicates how many proteins in total (in the network and in the background) have this term assigned.

2. The Strength is measure as Log10 (observed / expected) and describes how large the enrichment effect is. It’s the ratio between i) the number of proteins in your network that are annotated with a term and ii) the number of proteins that we expect to be annotated with this term in a random network of the same size.

3. False discovery rate describes how significant the enrichment is. Shown are p-values corrected for multiple testing within each category using the Benjamini–Hochberg procedure.

Table S9: Results from MalaCards database ^1^ for top 15 salient genes ranked according to LRI score. Gene’s cells are colored as aqua blue, olive green and purple for ‘No’, ‘Low’ and ‘High’ MIFTS score against CRC.

| **Sl No.** | **Salient Genes as Putative Biomarker** | **#Hits against Diseases** | **Hits against major cancer (other than CRC)** | **MIFTS score against CRC** | **MIFTS result against CRC** | **Reference / remarks** |
| --- | --- | --- | --- | --- | --- | --- |
| 1 | **MIR143HG** | 16 | - Very low score against Hepatocellular Carcinoma, Glioblastoma, Endometrial Cancer | - | NONE | - |
| 2 | **AMOTL1** | 52 | - High score against Meningioma.  - Moderate score against Cervical Cancer.  - Very low score against Hepatocellular Carcinoma, Glioblastoma, Glioma, Pancreatic Cancer, Esophageal Cancer, Acute Lymphoblastic Leukemia, **Breast Cancer**, Prostate Cancer, Lymphoma, Squamous Cell Carcinoma. | - | NONE | - |
| 3 | **ACTG2** | 113 | - High score against Gastrointestinal Stromal Tumor.  - Moderate score against Scrotum Neoplasm, Papillary Hidradenoma, Vulvar Sarcoma, Breast Myoepithelial Carcinoma.  - Very low score against Hepatocellular Carcinoma, **Breast Cancer**, Pancreatic Cancer, Prostate Cancer, Lung Cancer, Bladder Cancer, Osteogenic Sarcoma, Kidney Cancer | 0.16 | Yes (very low score) | ^2^ |
| 4 | **FILIP1** | 20 | - Moderate score against Glioblastoma.  - Very low score against **Breast Cancer**, Pancreatic Cancer, Ovarian Cancer, Lung Carcinoma. | - | NONE | - |
| 5 | **ARHGEF17** | 12 | - Very low score against Lung Cancer, Melanoma, **Breast Cancer**, Esophageal Cancer, Pancreatic Cancer, Neuroblastoma. | 0.215 | Yes (very low score) | ^3^ |
| 6 | **FAM219B** | 4 | - Very low score against **Breast Cancer**. | - | NONE | - |
| 7 | **ITPKB** | 69 | - Moderate score against Jaw Cancer, Maxillary Cancer.  - Very low score against Lung Cancer, **Breast Cancer**, Glioma, Pancreatic Cancer, Neuroblastoma, Lymphoma, Acute Myeloid Leukemia, Melanoma, Renal Cell Carcinoma, Adenocarcinoma, Embryonal Carcinoma, Renal Cell Carcinoma, Endometrial Cancer, Gastric Cancer, Chromophil Renal Cell Carcinoma. | 1.254 | Yes (very low score) | ^4^ |
| 8 | **TOP2A** | 301 | - High score against **Breast Cancer**, Lung Cancer, Gastric Cancer, Bladder Cancer, Neuroblastoma, Prostate Cancer, Ovarian Cancer, Retinoblastoma, Malignant Peripheral Nerve Sheath Tumor, Acute Lymphocytic Leukemia, Adenocarcinoma.  - Moderate score against Neoplasm, Cervical Cancer, Hepatocellular Carcinoma, Nonpapillary Renal Cell Carcinoma, Pancreatic Cancer.  - Very low score against Glial Tumor, Glioma, Glioblastoma, Leukemia, Esophageal Cancer, Leiomyoma, Endometrial Cancer, Neurofibroma, Gallbladder Cancer, Oligodendroglioma, Cholangiocarcinoma, Myeloma, Melanoma, Thymoma, Kidney Cancer, Meningioma, Medulloblastoma, Adenoma, Testicular Cancer, Brain Cancer, Anus Cancer, Biliary Tract Cancer, Pituitary Tumors, Thyroid Cancer, Lymphoma, Sarcoma, Carcinoma, Mesothelioma, Rhabdoid Cancer, Central Nervous System Cancer, Germ Cells Tumors, Hepatoblastoma, Hypopharynx Cancer. | 23.838 | Yes | ^5–9^ |
| 9 | **HAND1** | 87 | - Very low score against Thyroid Carcinoma, Medulloblastoma, Choriocarcinoma, **Breast Cancer**, Pancreatic Cancer, Gastric Cancer, Melanoma, Ovarian Cancer, Neuroblastoma, Testicular Yolk Sac Tumor, Endometrial Adenocarcinoma, Endodermal Sinus Tumor, Leukemia. | 0.359 | Yes (very low score) | ^10,11^ |
| 10 | **SERINC2** | 24 | - Very low score against Glioblastoma, Glial Tumor, Glioma, Lung Cancer, Adenocarcinoma, Ovarian Cancer, **Breast Cancer**, Pancreatic Cancer, Adenoid Cystic Carcinoma, Leukemia. | - | NONE | - |
| 11 | **TRAP1** | 144 | - Moderate score against Renal Cell Carcinoma, Ovarian Cancer, **Breast Cancer**, Glioblastoma, Glioma.  - Very low score against Lung Cancer, Ovarian Cancer, Adenocarcinoma, Squamous Cell Carcinoma, Hepatocellular Carcinoma, Pancreatic Cancer, Leukemia, Gastric Cancer, Neuroblastoma, Thyroid Carcinoma, Retinoblastoma, Prostate Cancer, Squamous Cell Carcinoma, Esophageal Cancer, Kidney Cancer, Lymphoma, Melanoma, Renal Cell Carcinoma, Nonpapillary, Bladder Cancer, Endometrial Cancer, Nasopharyngeal Carcinoma. | 1.29 | Yes (very low score) | ^12–14^ |
| 12 | **CAMSAP1** | 20 | - Moderate score against Laryngeal Benign Neoplasm.  - Very low score against Laryngeal Squamous Cell Carcinoma, Meningioma, Glioma, Oligodendroglioma, Pilocytic Astrocytoma, **Breast Cancer**, Ovarian Cancer, Pancreatic Cancer, T-Cell Lymphoma, Hepatocellular Carcinoma, Acute Lymphoblastic Leukemia, Prostate Cancer. | 0.222 | Yes (very low score) | ^15^ |
| 13 | **APOBR** | 25 | - Very low score against Acute Lymphoblastic Leukemia, Bladder Cancer, Glial Tumor, Glioma. | - | NONE | - |
| 14 | **PAG1** | 54 | - Very low score against Neuroblastoma, Larynx Cancer, Nasopharyngeal Carcinoma, **Breast Cancer**, Pancreatic Cancer, Leukemia, Lymphoma, Choriocarcinoma, Melanoma. | 0.551 | Yes (very low score) | Obscure result for Colorectal cancer |
| 15 | **MRPS9** | 6 | - Very low score against Lung Cancer, Adenocarcinoma, **Breast Cancer**, Pancreatic Cancer. | - | NONE | - |

**Figures S1 (for Figure 7):** Kaplan-Meier (KM) survival analysis of overall survival with high and low expression compared to the respective threshold median expression of AMOTL1 protein-coding genes in patients with colorectal cancer. The censored data are represented as vertical tick on the KM plot. **
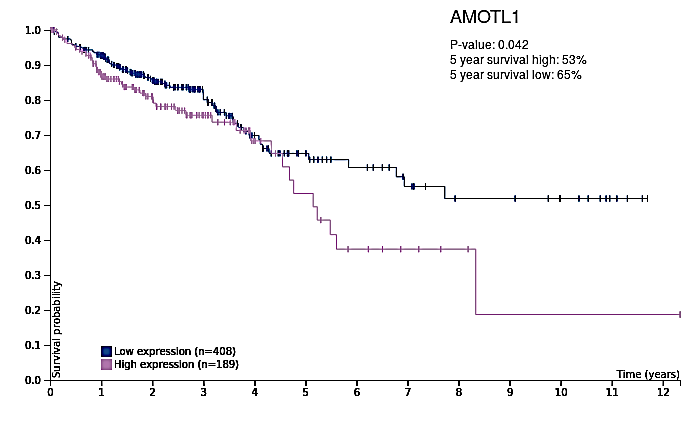
**

**Figures S2 (for Figure 7):** Kaplan-Meier (KM) survival analysis of overall survival with high and low expression compared to the respective threshold median expression of ACTG2 protein-coding genes in patients with colorectal cancer. The censored data are represented as vertical tick on the KM plot.
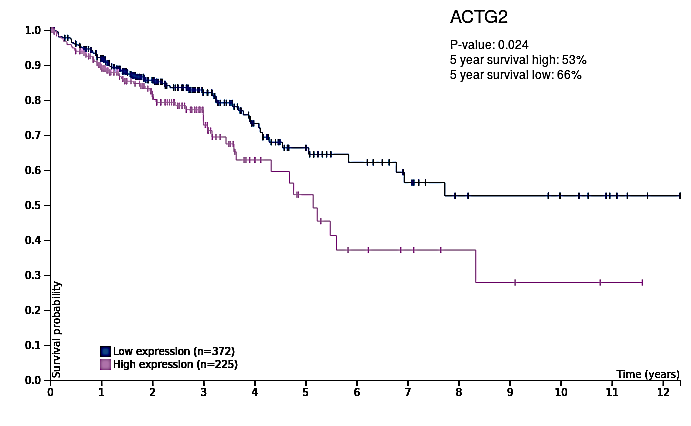


**Figures S3 (for Figure 7):** Kaplan-Meier (KM) survival analysis of overall survival with high and low expression compared to the respective threshold median expression of FILIP1 protein-coding genes in patients with colorectal cancer. The censored data are represented as vertical tick on the KM plot.
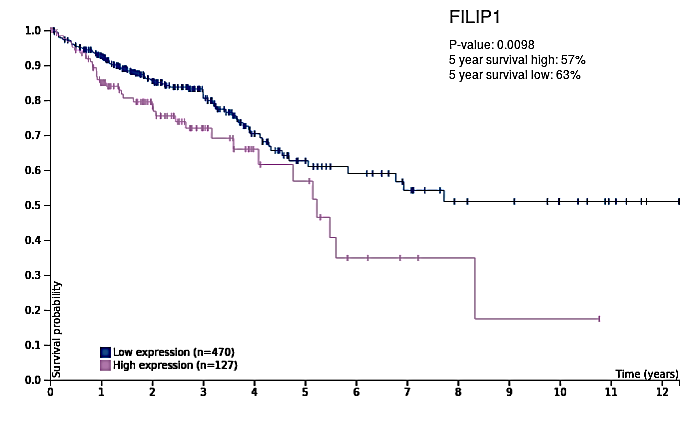


**Figures S4 (for Figure 7):** Kaplan-Meier (KM) survival analysis of overall survival with high and low expression compared to the respective threshold median expression of ARHGEF17 protein-coding genes in patients with colorectal cancer. The censored data are represented as vertical tick on the KM plot.
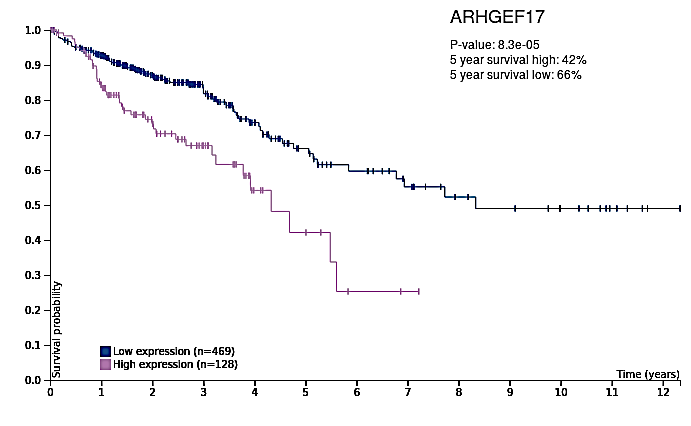


**Figures S5 (for Figure 7):** Kaplan-Meier (KM) survival analysis of overall survival with high and low expression compared to the respective threshold median expression of FAM219B protein-coding genes in patients with colorectal cancer. The censored data are represented as vertical tick on the KM plot.
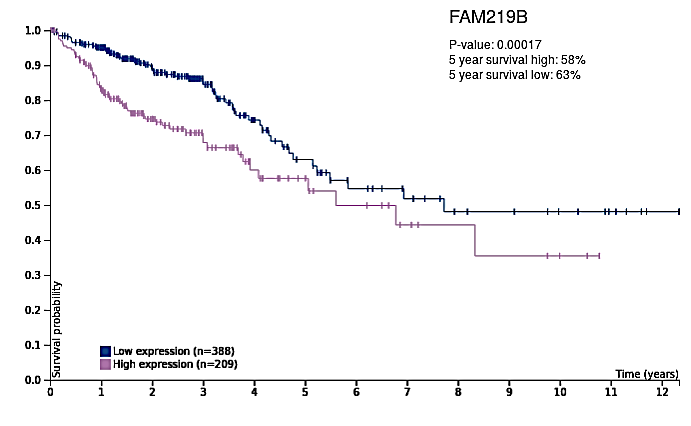


**Figures S6 (for Figure 7):** Kaplan-Meier (KM) survival analysis of overall survival with high and low expression compared to the respective threshold median expression of ITPKB protein-coding genes in patients with colorectal cancer. The censored data are represented as vertical tick on the KM plot.
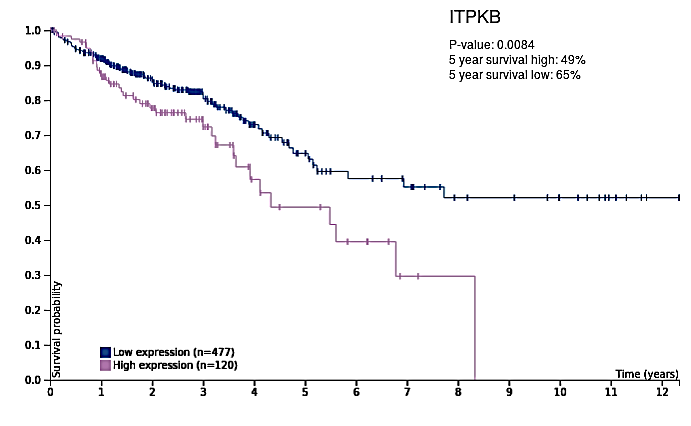


**Figures S7 (for Figure 7):** Kaplan-Meier (KM) survival analysis of overall survival with high and low expression compared to the respective threshold median expression of TOP2A protein-coding genes in patients with colorectal cancer. The censored data are represented as vertical tick on the KM plot.
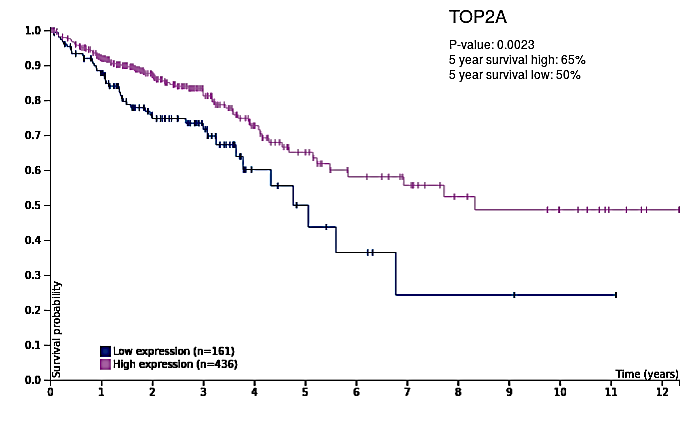


**Figures S8 (for Figure 7):** Kaplan-Meier (KM) survival analysis of overall survival with high and low expression compared to the respective threshold median expression of HAND1 protein-coding genes in patients with colorectal cancer. The censored data are represented as vertical tick on the KM plot.
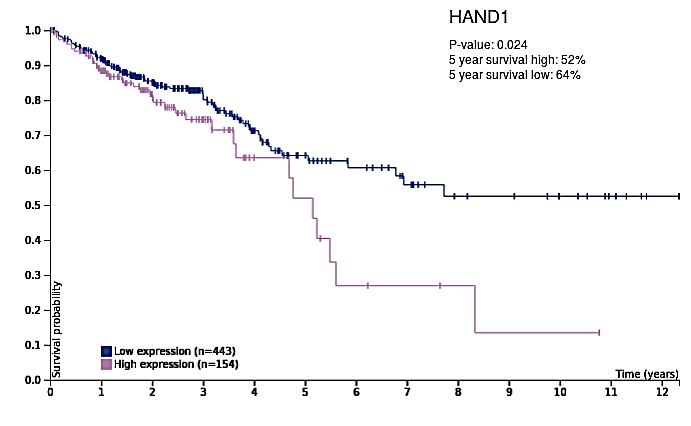


**Figures S9 (for Figure 7):** Kaplan-Meier (KM) survival analysis of overall survival with high and low expression compared to the respective threshold median expression of SERINC2 protein-coding genes in patients with colorectal cancer. The censored data are represented as vertical tick on the KM plot.
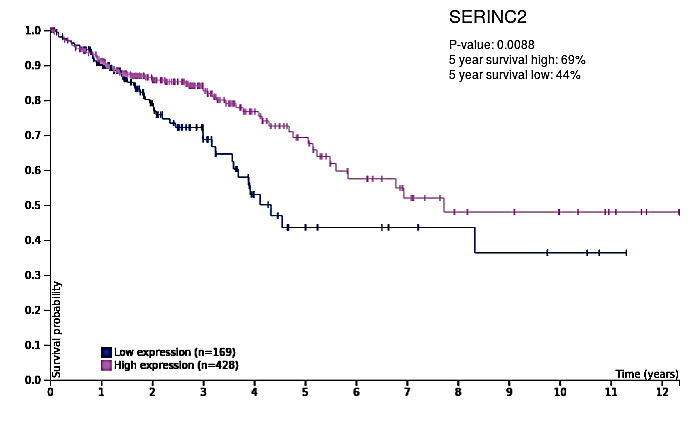


**Figures S10 (for Figure 7):** Kaplan-Meier (KM) survival analysis of overall survival with high and low expression compared to the respective threshold median expression of TRAP1 protein-coding genes in patients with colorectal cancer. The censored data are represented as vertical tick on the KM plot.
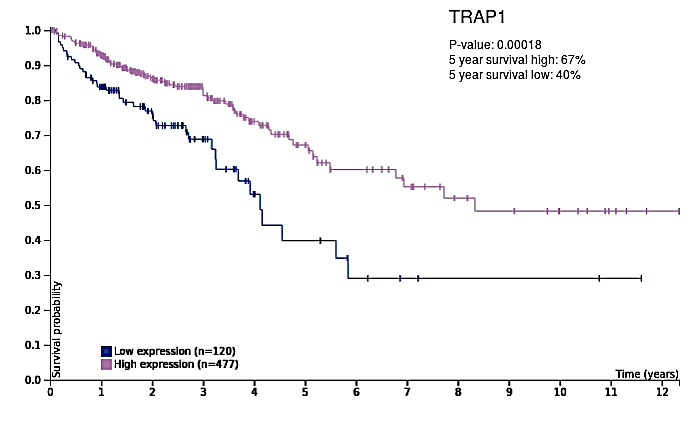


**References**

1. Rappaport, N. *et al.* MalaCards: An amalgamated human disease compendium with diverse clinical and genetic annotation and structured search. *Nucleic Acids Res.* **45**, D877–D887 (2017).

2. Zhao, B. *et al.* Identification of Potential Key Genes and Pathways in Early-Onset Colorectal Cancer Through Bioinformatics Analysis. *Cancer Control* **26**, (2019).

3. Fang, L. T. *et al.* Comprehensive genomic analyses of a metastatic colon cancer to the lung by whole exome sequencing and gene expression analysis. *Int. J. Oncol.* **44**, 211–21 (2014).

4. Li, B., Shi, C., Zhao, J. & Li, B. Long noncoding RNA CCAT1 functions as a ceRNA to antagonize the effect of miR-410 on the down-regulation of ITPKB in human HCT-116 and HCT-8 cells. *Oncotarget* **8**, 92855–92863 (2017).

5. Nygård, S. B. *et al.* Underpinning the repurposing of anthracyclines towards colorectal cancer: Assessment of topoisomerase II alpha gene copy number alterations in colorectal cancer. *Scand. J. Gastroenterol.* **48**, 1436–1443 (2013).

6. Gao, X. H. *et al.* ZNF148 modulates TOP2A expression and cell proliferation via ceRNA regulatory mechanism in colorectal cancer. *Medicine (Baltimore).* **96**, e5845 (2017).

7. Tarpgaard, L. S. *et al.* A phase II study of Epirubicin in oxaliplatin-resistant patients with metastatic colorectal cancer and TOP2A gene amplification. *BMC Cancer* **16**, 91 (2016).

8. Zhang, R., Xu, J., Zhao, J. & Bai, J. H. Proliferation and invasion of colon cancer cells are suppressed by knockdown of TOP2A. *J. Cell. Biochem.* **119**, 7256–7263 (2018).

9. Zhu, C. *et al.* UPF1 promotes chemoresistance to oxaliplatin through regulation of TOP2A activity and maintenance of stemness in colorectal cancer. *Cell Death Dis.* **12**, 519 (2021).

10. Tan, J. *et al.* Integrative epigenome analysis identifies a Polycomb-targeted differentiation program as a tumor-suppressor event epigenetically inactivated in colorectal cancer. *Cell Death Dis.* **5**, e1324 (2014).

11. Jin, B. *et al.* DNMT1 and DNMT3B modulate distinct polycomb-mediated histone modifications in colon cancer. *Cancer Res.* **69**, 7412–21 (2009).

12. Pak, M. G., Koh, H. J. & Roh, M. S. Clinicopathologic significance of TRAP1 expression in colorectal cancer: a large scale study of human colorectal adenocarcinoma tissues. *Diagn. Pathol.* **12**, 6 (2017).

13. Kim, K. *et al.* Differential expression of HSP90 isoforms and their correlations with clinicopathologic factors in patients with colorectal cancer. *Int. J. Clin. Exp. Pathol.* **12**, 978–986 (2019).

14. Shang, H. S. *et al.* Casticin induced apoptotic cell death and altered associated gene expression in human colon cancer colo 205 cells. *Environ. Toxicol.* **32**, 2041–2052 (2017).

15. Zhou, C. *et al.* circCAMSAP1 Promotes Tumor Growth in Colorectal Cancer via the miR-328-5p/E2F1 Axis. *Mol. Ther.* **28**, 914–928 (2020).
